# ConBind: motif-aware cross-species alignment for the identification of functional transcription factor binding sites

**DOI:** 10.1093/nar/gkv1518

**Published:** 2015-12-31

**Authors:** Stefan H. Lelieveld, Judith Schütte, Maurits J.J. Dijkstra, Punto Bawono, Sarah J. Kinston, Berthold Göttgens, Jaap Heringa, Nicola Bonzanni

**Affiliations:** 1 Centre for Integrative Bioinformatics VU, VU University Amsterdam, Amsterdam 1081 HV, The Netherlands; 2 Department of Human Genetics, Radboud Institute for Molecular Life Sciences, Radboud University Medical Center, Nijmegen 6525 GA, The Netherlands; 3 Department of Haematology, Wellcome Trust-MRC Cambridge Stem Cell Institute & Cambridge Institute for Medical Research, Cambridge University, Cambridge CB2 0XY, UK; 4 Klinik für Hämatologie, Universitätsklinik Essen 45147, Germany; 5 Computational Cancer Biology Group, Division of Molecular Carcinogenesis, The Netherlands Cancer Institute, Amsterdam 1066 CX, The Netherlands; 6 ENPICOM, Eindhoven 5632 CW, The Netherlands

## Abstract

Eukaryotic gene expression is regulated by transcription factors (TFs) binding to promoter as well as distal enhancers. TFs recognize short, but specific binding sites (TFBSs) that are located within the promoter and enhancer regions. Functionally relevant TFBSs are often highly conserved during evolution leaving a strong phylogenetic signal. While multiple sequence alignment (MSA) is a potent tool to detect the phylogenetic signal, the current MSA implementations are optimized to align the maximum number of identical nucleotides. This approach might result in the omission of conserved motifs that contain interchangeable nucleotides such as the ETS motif (IUPAC code: GGAW). Here, we introduce ConBind, a novel method to enhance alignment of short motifs, even if their mutual sequence similarity is only partial. ConBind improves the identification of conserved TFBSs by improving the alignment accuracy of TFBS families within orthologous DNA sequences. Functional validation of the Gfi1b + 13 enhancer reveals that ConBind identifies additional functionally important ETS binding sites that were missed by all other tested alignment tools. In addition to the analysis of known regulatory regions, our web tool is useful for the analysis of TFBSs on so far unknown DNA regions identified through ChIP-sequencing.

## INTRODUCTION

Accurate binding of transcription factors (TFs) to DNA is necessary for the normal functioning of all cell types. TFs bind to short (5–10 bp) DNA sequences known as DNA binding motifs or TF binding sites (TFBSs), and through interactions with the basic transcriptional machinery they control whether a gene is turned on or off. Functional regulatory DNA elements such as promoters and enhancers are often evolutionarily conserved; comparative DNA sequence analysis has therefore long been recognized as a powerful approach to both locate candidate regulatory regions, and also to pinpoint critical binding sites within such regions (1–3). Within the haematopoietic system, the TF Gfi1b (growth factor independence 1b) is expressed in haematopoietic stem cells as well as in common myeloid progenitors and it is essential for erythroid and megakaryocytic differentiation (4,5). Anguita *et al*. (6) identified a number of conserved non-coding elements (CNEs) containing multiple erythroid specific TFBSs through a multiple species sequence comparison approach. Three of these CNEs could be validated as haematopoietic enhancers in transgenic mouse assays (7), highlighting the importance of comparative DNA sequence analysis.

To study the transcriptional regulation of gene expression, it is not only necessary to determine conserved promoter or enhancer elements, but also to identify functional TFBSs within these regulatory elements. While *de novo* motif discovery methods such as MEME (8) and RSAT (9) are commonly used for the prediction of novel TF binding motifs within regulatory regions, ConBind is designed to simplify and accelerate hypothesis-driven research, helping biologists prioritizing experiments and validations for the known TFBSs most likely to be functional. Usually, phylogenetic footprinting methods (10,11), are employed to predict whether a certain putative TFBS is functional or not. The underlying principle behind the phylogenetic footprinting technique is that functional sequence motifs tend to be more conserved between species than the non-functional sequence motifs. ConBind improves on current phylogenetic footprinting methods by using relevant biological information (i.e. TF binding motifs) to produce motif-aware alignments that increase the identification of conserved TFBSs.

Recent improvements in genome-wide sequencing approaches have resulted in an explosion of completely sequenced genomes including viruses, bacteria and eukaryotes. This major increase in the availability of sequenced genomes has allowed for more widespread use of the phylogenetic footprinting method, as it relies heavily on sequence alignment to assess the conservation of the regulatory elements. A phylogenetic footprinting analysis commonly starts with a query sequence of an organism of interest, followed by collection of sequences, which are orthologous to this query sequence. Finally, the query sequence and its orthologs are aligned together using a multiple sequence alignment (MSA) algorithm of choice. TFBSs that are present in the query sequence are deemed functional when they are conserved in the alignment of multiple species.

A biological sequence alignment, pairwise or multiple (alignment of three or more sequences), is obtained by inserting gaps into sequences such that all sequences in the alignment have the same length L. The goal of the sequence alignment technique is to arrange the N input sequences into a matrix of N rows and L columns in such a way that best represents the evolutionary relationships among these sequences. Sequence alignment methods are commonly employed to infer conserved (functional) sequence elements. Several tools are available for generating pairwise as well as MSAs such as ClustalW2 (12), ClustalOmega (13), Praline (14), MUSCLE (15), T-Coffee (16) and MAFFT (10).

The currently available tools for identifying functional TFBSs, such as TOUCAN 2 (11), ConSite (17) and rVISTA 2.0 (18) make use of a sequence alignment algorithm, which applies a generic scoring scheme aimed to maximize the number of matching nucleotides in the aligned sequences. Hence, these methods sometimes prevent correct alignment of conserved TFBSs, especially for motifs with low specificity, for example the well described EBOX (CANNTG) or ETS (GGAW) motifs. We present here ConBind, a web-based online tool that addresses these shortcomings. An intuitive interface allows the user to input the DNA sequence or genomic coordinates from one species and then select multiple species from a list that will be used for the generation of the MSA(s). Finally, a number of DNA sequence motifs can be selected from the provided list or the sequence of TF binding motifs of interest can be added to the input information. The selected motifs will be employed to optimize the sequence alignment based on TFBS conservation. The output consists of MSAs with highlighted TFBSs, accessible online for consultation or downloadable in FASTA, JALVIEW (19), MSF, RTF or XML format for further processing. Taken together, this tool is a useful resource for researchers interested in gene regulation by DNA binding proteins.

## MATERIALS AND METHODS

**Figure 1.**
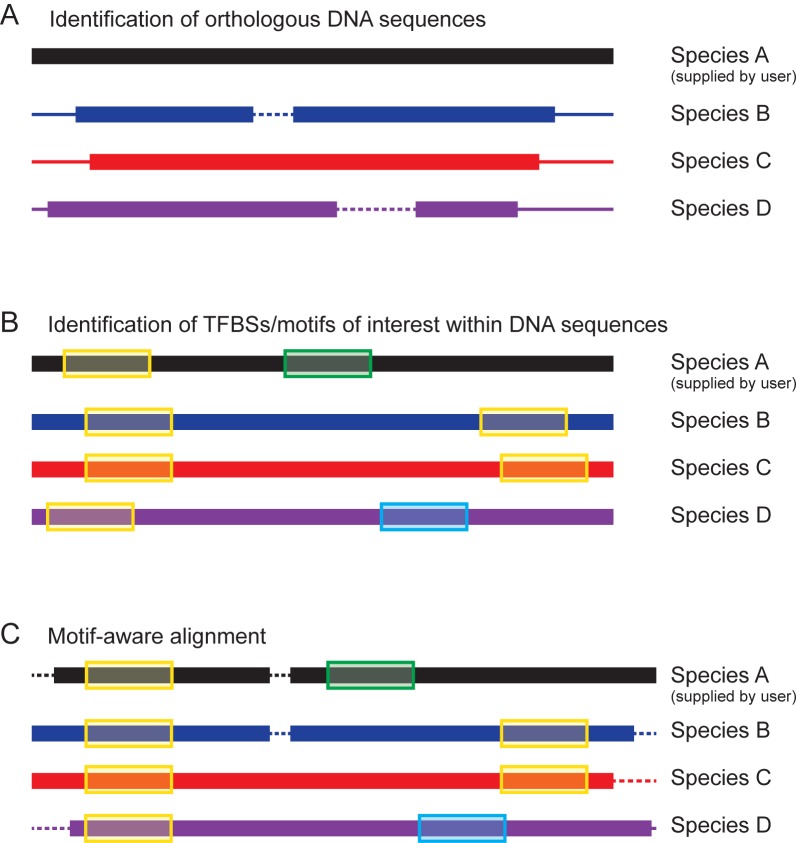
Schematic diagram of the ConBind pipeline. (**A**) Identification of suitable orthologous DNA sequences (species B, C and D) for the user-supplied sequence (species A). Core orthologous subsequences are found using BLAST. (**B**) The orthologous DNA sequences are extended to match the length of the region of interest (black) and TFBSs of interest (supplied by user) are identified (**C**) Motif-aware alignment of the orthologous DNA sequences by using the candidate TFBSs information to optimize the MSA. Dashed lines represent gaps in the alignment.

We implemented a phylogenetic footprinting pipeline as a web application named ConBind available for non-commercial use at http://www.conbind.org. Our pipeline is based on three steps: (i) identification of suitable orthologous regions, (ii) motif-aware alignment of the orthologous sequences and finally (iii) visualization of conserved TFBSs (see Figure 1).

### Identification of suitable orthologous sequences

Given the chromosomal coordinates (i.e. assembly name, chromosome number, start and end position) of the regulatory region of interest, the corresponding sequence is extracted from the genome. Alternatively, the DNA sequence of interest can be provided as input. The target sequence is then used as a query to find orthologous regions running BLASTn on the genomes of the species selected by the user. The Smith–Waterman (local alignment) algorithm used by BLASTn identifies only the high similarity core subsequence. Therefore, in order to better match the size of the query region, we extended starting and ending chromosomal positions of each core subsequence to match the length of the query. The extended chromosomal coordinates are then used to retrieve the nucleotide sequences of the orthologous regions. *E*-values from BLAST searches are stored and reported as part of the output file for the user to assess the quality of the orthologous sequence retrieved.

### Motif-aware alignment of the orthologous sequences

**Figure 2.**
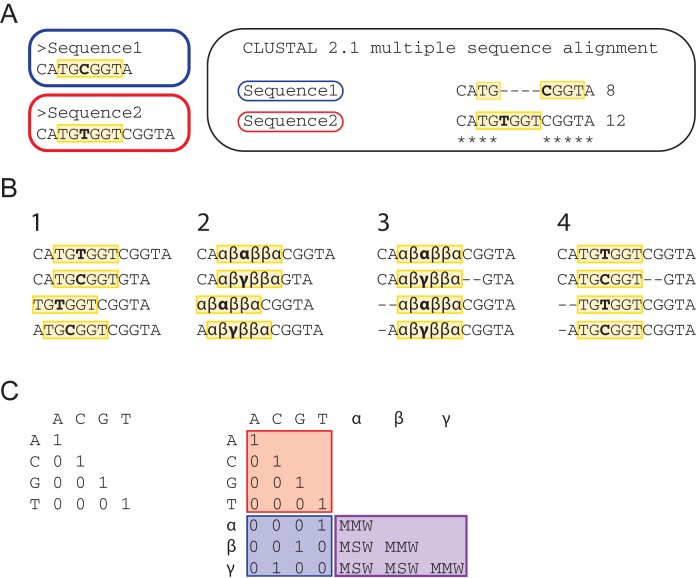
Generation of motif-aware alignment. (**A**) TFBSs of the same motif family that are comprised of different nucleotides are often not aligned with each other by current MSA algorithms such as ClustalW2. Both, Sequence1 and Sequence2, contain the RUNT DNA binding motif (highlighted in yellow), but the sequence differs by one nucleotide (bold). On the right, the alignment produced by ClustalW2 is shown. Gaps are introduced inside the motif in order to maximize the overall alignment score. (**B**) Step-wise substitution of nucleotides: (1) The locations of the RUNT motif (yellow) are marked in four different sequences; (2) The letter for each nucleotide embodied in a motif is replaced by a new symbol carrying information about the original base type and the motif family; (3) The MSA is computed using the extended weight matrix (see panel **C**); (4) The symbols are replaced with the original base letters on the aligned sequences. (C) Left: the default identity matrix rewards the alignment of equal nucleotides per column, irrespective of their biological context. Right: the original identity matrix (red) is extended to take into account information about TF binding motifs. Nucleotides embodied in a motif are rewarded in a similar way to the default identity matrix when they match the original nucleotides (blue). Alignment of bases of the same motif family that differ between two or more sequences are rewarded by a MMW (Motif Match Weight) or MSW (Motif Mismatch Weight) depending on the original nucleotide.

Current MSA methods were developed under the assumption that nucleotide occurrences are randomly distributed and independent from neighboring bases. Therefore, identity matrices (i.e. matrices with a score of one on the main diagonal only) are used as weight matrices to compute nucleotide alignments. Such matrices reward only the alignment of nucleotide of the same type. However, aligning each individual nucleotide without taking information of neighboring nucleotides into account is not sufficient for the alignment of those TFBSs that can contain variable nucleotides within their core sequences such as ETS motifs with the IUPAC consensus sequence GGAW (20), STAT5 binding sites with the IUPAC consensus sequence TTCYNRGAA (21) or RUNT motifs with the IUPAC consensus sequence TGYGGT (22). For instance, TFs that recognize the RUNT motif (e.g. RUNX1) can bind to both TGCGGT and TGTGGT sequences. In a motif-aware alignment, both motif possibilities would ideally align equally well; hence TGCGGT would align not only to itself, but also to TGTGGT. However, current MSA algorithms are optimized to maximize the overall alignment score based on the identity matrix and therefore do not account for biological information such as known TFBSs. As a result, standard MSA methods can misalign biologically conserved TFBSs as shown in Figure 2A.

In order to increase the identification and prediction accuracy of conserved TFBSs, we enhanced the MSA step by integrating information about the TF motif families specified by the user (e.g. ETS, RUNT, GATA); where a motif family is a user defined non-empty set of consensus sequences. The MSA rewards in this case the alignment of two (possibly different) nucleotides belonging to the same motif family as described above. Therefore, it is necessary to distinguish between nucleotides that are part of a DNA binding motif and those that are not. In order to achieve this, the original nucleotide sequence alphabet (A, C, G, T) needs to be complemented with new symbols (i.e. letter different than A, C, G, T) that contain information about the nucleotide type as well as the affiliation to a specific motif family, i.e. each nucleotide type/family combination will be represented by different symbol. The new symbols therefore replace the original nucleotide characters in case the nucleotides are part of motif sequences (see Figure 2B). Since motif patterns of different families can overlap, a single position on a sequence can belong to multiple motif families. As the replacement symbol will have to carry information about all overlapping families, it will be different form the symbol used for the same base in each of the various families. For instance, each guanine residing in an overlap between an EBOX and a RUNT motif will be assigned the replacement symbol **α**, while each guanine belonging only to an EBOX motif will have the symbol **β** and the ones belonging only to a RUNT motif will have the symbol **γ**. Using this strategy, it is possible to score higher the alignment of **α** with **β** or **γ**, than the alignment of **β** with **γ**.

Once the aforementioned symbol substitution is complete, the default weight matrix (i.e. the identity matrix over A, C, G, T) has to be extended with the new symbols (Figure 2C). The extended matrix assigns weights to the following eight combinations of pairwise comparisons: (1) two nucleotides letters of the same base (e.g. G matching G), (2) two nucleotides letters of different bases (e.g. G matching C), (3) a nucleotide and a replacement symbol corresponding to the same base (e.g. T matching **α**), (4) a nucleotide and a symbol corresponding to different bases (e.g. G matching **α**, where **α** replaces a base different than G), (5) two identical symbols (e.g. **α** matching another **α**), (6) two different symbols belonging to the same motif family and replacing different bases (e.g. **α** and **β**, where the first correspond to a G and the latter to an A of the GATA motif), (7) two different symbols belonging to different motif families but replacing the same base (e.g. **α** and **γ** where the first correspond to a G in a GATA motif and the latter to a G in an EBOX motif) and finally (8) two different symbols belonging to different motif families and replacing different bases (e.g. **α** and **δ** where the first correspond to a G in a GATA motif and the latter to a T in a RUNT motif).

In order to produce a motif-aware alignment the traditional weight matrix has to be extended with additional symbols and weight assignments. The weights allocated to each of the eight pairwise comparisons are shown in Table 1. Briefly, a weight of 1 is assigned when the same nucleotides are aligned (case 1) as well as when a nucleotide or symbol are aligned that represent the same base (cases 3 and 7). A weight of 0 is assigned when nucleotides and/or symbols do not match (cases 2, 4 and 8). The score in the weight matrix has to be increased for those cases in which symbols within the same TFBS are compared (cases 5 and 6), so that TFBS are preferentially aligned over any random DNA sequence. This up-weighting rewards the alignment of bases belonging to the same motif family but with different nucleotide compositions such as GGAA and GGAT for the ETS motif with the IUPAC code GGAW.

**Table 1.** Matching nucleotides letters and enriched symbols give rise to eight possible cases

Weight	Case
0	(2) Two nucleotide letters of different bases
	(4) A nucleotide letter and a symbol corresponding to different bases
	(6) Two different symbols belonging to different motif families and shadowing different bases
1	(1) Two nucleotides letters of the same base
	(3) A nucleotide letter and a symbols corresponding to the same base
	(5) Two different symbols belonging to different motif families but shadowing the same base
MMW ( = 3)	(8) Two identical symbols
MSW ( = 1)	(7) Two different symbols belonging to the same motif family and shadowing different bases

Cases 2, 4 and 6 correspond to the default mismatch case with a weight of 0, i.e. when two different nucleotide bases are compared. Cases 1, 3 and 5 correspond to the default match cases, i.e. when two identical nucleotides bases are compared. Case 7 correspond to match two identical nucleotides that belong to a motif sequence of the same family. Case 8 correspond to match two different nucleotide bases belonging to a motif sequence of the same family. The weight for case 8 (motif match weight or MMW) and case 7 (motif mismatch weight or MSW) were set to 3 and 1 respectively.

The choice of the motif match weight (MMW) (case 5) and motif mismatch weight (MSW) (case 6) is extremely important as these two weights are responsible for the subtle equilibrium between overall alignment accuracy and a favored alignment of TFBSs. On the one hand, a weight that is too small will produce a very similar alignment to the currently available tools in which the maximum number of nucleotides will be aligned without allowing the alignment of different bases embodied in the same motifs. On the other hand, too heavy weights will produce low accuracy because the MSA algorithm will force TFBSs to be aligned although they are far apart, resulting in the introduction of an unrealistic amount of gaps. To choose the most appropriate weights, we trained ConBind using a set of experimentally validated TFBSs on validated promoter and enhancer regions as described in the Supplementary Data.

Any alignment tool that can accept custom weight matrices and sequences containing the replaced symbols can be used to generate a motif-aware MSA. Importantly, the chosen MSA tool must support a wide range of characters beside the original four letters (i.e. A, C, G, T). The more symbols are supported the more motif families can be specified by the user. In our pipeline we employed Praline (default settings) (23), which, in its new implementation, supports the whole UNICODE character set representing more than 110 000 characters. After the MSA has been generated by Praline using the enriched symbols and custom scoring-matrix generated by ConBind, the symbols are finally replaced with the original nucleotide letters. The pseudo code of the algorithm describe above is shown in Supplementary Data.

### Visualization of motif-aware alignment

The final alignments are presented to the user online. Motif families are highlighted on the aligned sequences using different colors to facilitate identification and assessment of conservation. Each column in the alignment is annotated with a sum-of-pairs score (24) visualized as a bar with a gradient from white (score 0) to blue (score 1). This score can help to select TFBSs for further validation because conserved TFBSs in high score sub sequences are more likely to be functional. Alternatively, it is possible to submit alignments to ConBind and retrieve the results using a RESTful API. The programmatic API access is particularly convenient to integrate ConBind with other tools. A detailed documentation of the ConBind API is available on the ConBind website.

### Luciferase reporter assays

The Gfi1b + 13 enhancer was amplified from mouse genomic DNA using the following primers: taaggatccCAGGTGCTAGATCCCGTCAT (forward) and taagtcgacTTCCCTCTGGATGTCTGTGG (reverse). Mutant DNA fragments were generated using standard recombinant DNA techniques or were obtained from GeneArt^®^ by Life Technologies (see Supplementary Data for details). The enhancer was cloned into pGL2 promoter (Promega) using BamHI and SalI restriction enzymes. 416b cells (murine myeloid progenitor cell line) were transfected by electroporation (220V, 900μFarad) with the relevant enhancer constructs and a neomycin containing control vector. Experiments were performed in triplicates and each experiment contains at least three technical replicates. The luciferase activity of stably transfected cells was determined using the FLUOstar OPTIMA luminometer from BMG LABTECH. To compare the wild-type construct with the empty vector or the mutant constructs, *t*-tests (two-tailed, homoscedastic) were applied to the values of each individual experiment using the ttest function in Excel (Microsoft Office). The *P*-values were then combined using the Fisher's method in order to obtain an overall p-value for each comparison. The Fisher's method does not take into account the effect direction. In those cases, where the effect direction is different between experiments (Gfi1b + 13_Ebox, Gfi1b + 13_Ets1–2), Stouffer's z-score was calculated.

## RESULTS

### Performance assessment and comparison

Assuming that functional TFBSs are generally more conserved than non-functional binding sites, then the prediction of functional TFBSs relies on the proficiency of MSA methods to correctly identify conserved TFBSs. We tested the performance of ConBind in identifying conserved TFBSs using a set of regulatory regions for which functional TFBSs where previously experimentally validated. This set includes 14 previously published (6,25–31) regulatory regions (nine mouse and five human) resulting in a total of 59 experimentally validated TFBSs belonging to 15 motif families (i.e. ETS, GATA, GFI1, MEIS, SOX, YBOX, SP1F, SRE, VTBF, GC-box, GRH, HOX, DMTF, ZBPF and P53F). Importantly, none of the regions used for validation has been used during parameter estimation. Using ConBind, we aligned each regulatory region of one species with homolog regions from seven other species including *Homo sapiens, Mus musculus, Bos taurus, Canis lupus familiaris, Loxodonta africana, Monodelphis domestica, Sarcophilus harrisii* and *Ornithorhynchus anatinus*.

Since it is unlikely to experimentally test (and report) a binding site if it is not conserved, while other binding sites in the same enhancer show strong conservation, the benchmark does not contain true negatives. Therefore, the alignments produced by ConBind were evaluated using the Cost-Effectiveness scores (32). The Effectiveness score measures the conservation of each experimentally validated TFBS. This score thus counts the number of sequences (i.e. species) for which each TFBS appears in the same position in the alignment. The score can be represented as:

\begin{equation*} {\rm Effectiveness} = \frac{{\sum\nolimits_i^m {\frac{{n(T_i )}}{H}} }}{m}, \end{equation*}

where *m* is the total number of experimentally validated TFBSs, *H* the total number of sequences used in the alignment and *n*(*T_i_*) is the number of sequences in which a TFBS *T_i_* appears at the same position in the alignment. Hence, the value for Effectiveness goes toward 1 when more experimentally validated TFBSs appear to be conserved in the alignment.

However, the Effectiveness score alone is not sufficient to fully capture the performance of a MSA method. In fact, it is possible to forcefully align biologically unrelated TFBSs and obtain an artificial increase in Effectiveness score. For instance, the Effectiveness score can be maximized by inserting an unreasonable number of gaps in order to align TFBSs that are located far apart from each other, which results in an unrealistic and biologically meaningless alignment. It is therefore crucial to score, not only the TFBS alignment, but also the overall alignment. Considering both, it is possible to penalize and prevent nonsensical alignments by computing the sum-of-pairs score (33) over the alignments. Intuitively, this score represents the cost (in terms of overall alignment quality) that is paid to achieve certain Effectiveness. The Cost score can be written as:

\begin{equation*} {\rm Cost} = 1 - {\rm Sum - of - pairs - score}. \end{equation*}

**Figure 3.**
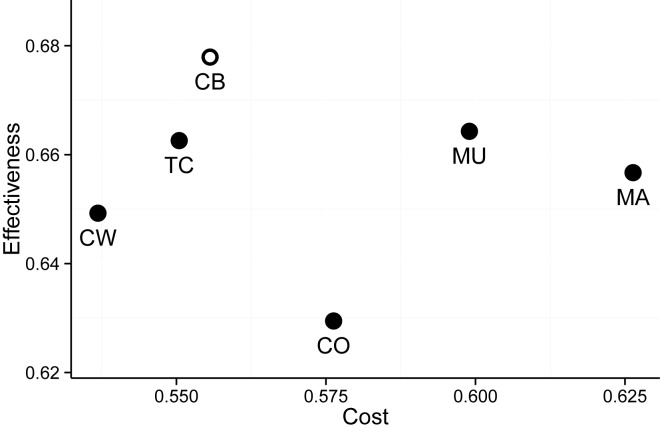
Cost-effectiveness plane showing the performances of ConBind (CB) versus five other popular MSA methods, ClustalW2 (CW), T-Coffee (TC), ClustalOmega (CO), MUSCLE (MU) and MAFFT (MA). Effectiveness and Cost were computed using a set of 50 experimentally validated TFBSs. The method that performs best for the identification of functional TFBSs is ConBind with the highest Effectiveness and higher Effectiveness/Cost ratio than other MSA algorithms.

The greater the Cost, the worse is the overall alignment quality. Given the 59 aforementioned TFBSs, we computed the Cost-Effectiveness for ConBind and other five popular MSA methods: ClustalW2 (34), ClustalOmega (13), MUSCLE (15), MAFFT (10) and T-Coffee (16) The Cost-Effectiveness for each method was computed over an identical set of sequences (retrieved via BLAST) using the default settings of the method respectively. The resulting Cost-Effectiveness plane (32) is shown in Figure 3. ConBind achieves the best Effectiveness at a Cost lower than most of the other MSA algorithms. This result reflects the fact that ConBind aligns an equal or greater number of species for conserved TFBSs.

Examining the alignments produced, it is noticeable that most methods are able to correctly align TFBSs when they are embedded in highly conserved regulatory regions. This is due to the fact that the positioning of the TFBSs in the alignment is constrained by the highly conserved surrounding fragments. Thus, the benefits of using ConBind are even more evident in fragments of regulatory regions, which are not so highly conserved. To show such benefits, we decided to compare the alignment produced by ConBind and ClustalW2 for the Gfi1b enhancer that is located 13 kb downstream of the ATG start codon, also known as Gfi1b + 13 or Gfi1b CNE + 1, and validate *in vitro* the functionality of the conserved TFBSs.

### Functional TFBSs within Gfi1b + 13 identified using ConBind

**Figure 4.**
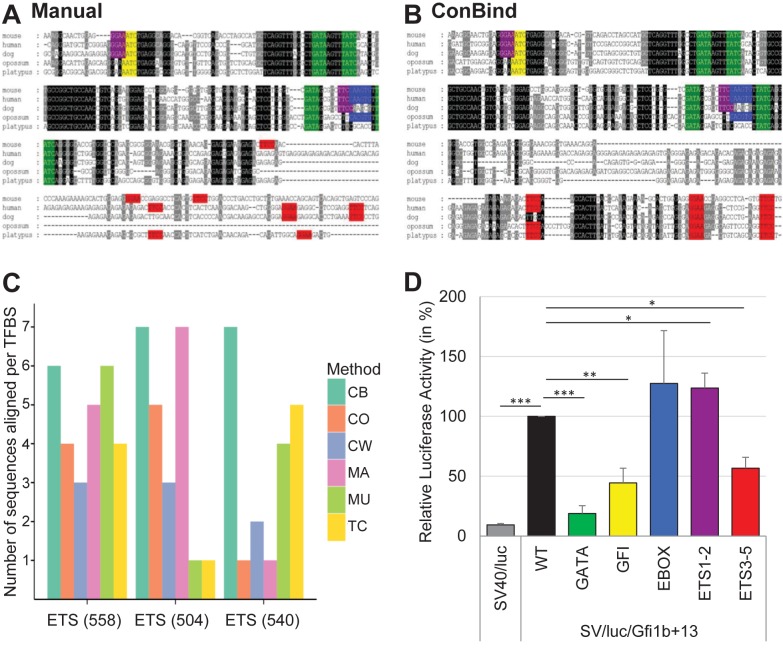
Identification of functional TFBSs in the Gfi1b + 13 enhancer. (**A**) Manual identification of functional TFBSs in the Gfi1b + 13 enhancer was performed as follows: (i) LiftOver of the mouse DNA sequence (mm9) to human (hg19), dog (canFam2), opossum (monDom5) and platypus (ornAna1) using UCSC (40). (ii) Alignment using ClustalW2 (12). (iii) Scoring conservation using GeneDoc (41). (iv) Manual search for TFBSs using Microsoft Word. ETS binding sites are shown in purple and pink, GFI binding sites in yellow, GATA motifs in green and EBOX motifs in blue. (**B**) Alignment of the Gfi1b + 13 enhancer using ConBind. Input data: Gfi1b+13 co-ordinates; (−)-strand; assembly: mm9, motifs: EBOX, ETS, GATA, GFI; species: human, dog, opossum, platypus. Output file was saved as msf-format in order to display conservation similarly to the ClustalW2 (12) alignment. The same genomic region (chr2:28,602,086–28,602,736, mm10) has been used for the manual alignment (A) as well as for the alignment using ConBind (B), but only the most conserved part of the enhancer is shown. Color scheme as in (A). (**C**) Comparison of different MSA methods for identification of the three ETS binding sites on the 3′ end of the Gfi1b + 13 enhancer. Annotated in parenthesis are the positions of TFBSs (in bp) relative to the start of the enhancer. Each bar represents a MSA method: ConBind (CB), ClustalW2 (CW), T-Coffee (TC), ClustalOmega (CO), MUSCLE (MU) and MAFFT (MA). The height of the bar shows the number of species aligned by each MSA method for each binding site (maximum of seven species). (**D**) Luciferase reporter assay in stably transfected 416b cells. All TFBSs of one motif family, e.g. all GATA motifs, were mutated at the same time by single nucleotide changes within each motif. The results are shown relative to the luciferase activity of the wild-type (WT) enhancer. Color scheme as in (A). *t*-test *P*-values: * ≤0.05, ** ≤0.01, *** ≤0.001. The exact *P*-values are as follows: SV40/luc = 6.69E-18; SV/luc/Gfi1b + 13_Gata = 3.25E-06; SV/luc/Gfi1b + 13_Gfi1 = 0.0027; SV/luc/Gfi1b + 13_Ebox = 0.812; SV/luc/Gfi1b + 13_1–2 = 0.014; SV/luc/Gfi1b + 13_Ets3–5 = 0.013.

The ClustalW2 alignment of mouse, human, dog, opossum and platypus sequences for the Gfi1b + 13 enhancer followed by a manual search (using the search function in Microsoft Word) for conserved TFBSs identified four highly conserved GATA motifs, one highly conserved GFI motif, one conserved EBOX motif and two conserved ETS binding sites, identical to what has previously been shown by Anguita *et al*. (6), but the 3′ end of the enhancer seemed to be only poorly conserved (see Figure 4A). In comparison, the MSA that was generated using ConBind shows similarities, but also differences (see Figure 4B). Firstly, ConBind recovers all conserved TFBSs highlighted in the ClustalW2 alignment. More importantly, by introducing gaps at the 3′ end of the mouse sequence, at the expense of a slightly decreased overall alignment score, three additional ETS binding sites could be identified (Figure 4B). These three ETS motifs were not or only partially found using various other methods (Figure 4C).

Expression of Gfi1b is regulated through binding of various haematopoietic TFs including Scl, E2A, Gata1 and Gfi1b itself to its promoter as well as four downstream regulatory elements, including the Gfi1b + 13 enhancer (6,31). Because binding alone does not indicate which effect the various TFs have on gene expression, we have performed luciferase reporter assays of wild-type and mutant versions of the Gfi1b + 13 enhancer (Figure 4D). Compared to the pGL2 promoter control vector, the Gfi1b + 13 enhancer is highly active (10-fold increase in luciferase activity). Whereas mutations of the highly conserved GATA or GFI binding sites, show a significant decrease in luciferase activity, the mutation of the less conserved EBOX motif does not show a significant effect on luciferase activity, therefore indicating that TFs binding to GATA and GFI motifs are important for the activation of Gfi1b gene expression. Interestingly, the mutation of the two ETS binding sites of the Gfi1b + 13 enhancer (ETS1–2) identified through the alignment with ClustalW2 slightly increases the luciferase activity compared to the wild-type, but mutation of the additional three ETS binding sites found using ConBind (ETS3–5) decreases the luciferase activity by almost 50% compared to the wild-type enhancer. These results clearly underline the functional relevance of the three ETS motifs identified by ConBind and missed by ClustalW2.

Additionally, detailed inspection of the hematopoietic active Lmo2–75 enhancer (35) reveals that the enhancer is comprised of two smaller sub-regions that are bound by a number of TFs in the hematopoietic progenitor cell line HPC7 (Supplementary Figure S4A). Analyzing these two sub-regions in luciferase reporter assays demonstrates that both sub-regions are transcriptionally active on their own (Supplementary Figure S4B). Importantly, ConBind was able to align the orthologous sequences for mouse, human, dog, opossum and platypus in a way that resulted in the identification of several conserved TFBSs within both sub-regions of the Lmo2–75 enhancer (Supplementary Figure S4C). In contrast, the manual alignment using ClustalW did not show any conserved TFBSs within sub-region 1 and fewer TFBSs within sub-region 2 (Supplementary Figure S4D). Furthermore, the alignment of the Lmo2–75 enhancer using ConBind highlights ConBind's ability to not only align perfectly matching TFBSs, but also those TFBSs that belong to the same motif family, such as TTCC and ATCC for the ETS motif or CAGATG and CAGGTG for the EBOX motif. As ChIP-Seq experiments show TF binding to both regions, it is likely that the conserved TFBS identified by ConBind are also functional.

## DISCUSSION

Gene expression is regulated through binding of TFs to promoter and enhancer regions. These regulatory regions are generally highly conserved throughout evolution. As a consequence, those TFBSs that are functionally important are also likely to be conserved between species. In order to identify conserved TFBSs, it is necessary to use a number of publicly available online tools in a step-wise manner. Due to the various steps involved, this manual evaluation of multi-species alignment to identify regulatory regions is very time-consuming. Firstly, the orthologous DNA sequences have to be identified and saved in such a way that MSA tools such as ClustalW2 can align the sequences. The output file subsequently needs to be modified in order to highlight conservation and finally, the TFBSs of interest have to be manually searched for by for example using Microsoft Word. ConBind greatly reduces the hands-on time needed for the generation of MSAs highlighting TFBSs. The user needs to provide the following data: (i) the chromosomal coordinates or DNA sequence of the region of interest, (ii) a name for this region, (iii) the strand information, (iv) the genome build, (v) the TF binding motifs of interest and (vi) the species that will be compared. It is optional to provide an email address, which will be used to send an email with the results as soon as the alignment is ready. This has the advantage that the webpage can be closed while the program is running without losing the output data. Furthermore, ConBind exposes a RESTful API interface, which allows to programmatically access (i.e. without using the web user interface) and integrate ConBind in other analysis workflows and tools.

ConBind not only reduces the time to generate MSAs with highlighted TFBSs, but it also improves the alignment of conserved TFBSs. Traditional alignment tools focus on the alignment of the maximum number of nucleotides in order to increase the overall alignment score. In contrast, ConBind was developed to identify a higher number of conserved TFBSs, which might play a functional role in regulation of gene expression, without excessively compromising the overall alignment score. This balance has been carefully analyzed by comparing different Cost and Effectiveness scores based on previously published datasets (Figure 3) (25–27,36–38). In order to verify the improved alignment software, we have tested conserved TFBSs within the Gfi1b + 13 enhancer. Importantly, the usage of ConBind led to the identification of the same conserved binding motifs as the alignment generated with ClustalW2 followed by a manual search for conserved TFBSs. But by introducing a number of gaps into the mouse sequence and therefore reducing the overall alignment score for this region, three additional ETS binding sites could be identified at the 3′ end of the sequence. Functional validation in luciferase reporter assays showed that these ETS sites are indeed important for the regulation of luciferase activity (Figure 4).

The availability of an increased amount of ChIP-Sequencing and the drastic decrease of full genome sequencing costs demand reliable and efficient methods for the characterization of regulatory regions extracted from these data. For instance, the recently developed compendium of haematopoietic ChIP-sequencing samples is a rich source for the identification of so far unknown regulatory elements (39). Although, *de novo* motif discovery across the whole dataset gives insights into the underlying regulatory mechanisms, it is still necessary to validate the findings in detail at the gene loci of interest. Here, ConBind can facilitate the identification of candidate regulatory regions for further analysis, as it can be easily tested if and how many conserved TFBSs are present within the selected candidate DNA regions.

Thus, ConBind not only simplifies and improves the identification of conserved TFBSs through the generation of MSAs incorporating motif information, but also helps to interpret TF binding events identified through ChIP-sequencing.

## Supplementary Material

SUPPLEMENTARY DATA
