# Supplementary material for: ConBind: motif-aware cross-species alignment for the identification of functional transcription factor binding sites
Source: Nucleic Acids Res. 2015 Dec 31;44(8):e72. doi: 10.1093/nar/gkv1518 (PMC4856970; doi:10.1093/nar/gkv1518)
Supplement: SUPPLEMENTARY DATA [file supp_44_8_e72__index.html]

ConBind: motif-aware cross-species alignment for the identification of functional transcription factor binding sites — SUPPLEMENTARY DATA 

# ConBind: motif-aware cross-species alignment for the identification of functional transcription factor binding sites

## SUPPLEMENTARY DATA

- SUPPLEMENTARY DATA
