# Supplementary material for: ConBind: motif-aware cross-species alignment for the identification of functional transcription factor binding sites
Source: Nucleic Acids Res. 2015 Dec 31;44(8):e72. doi: 10.1093/nar/gkv1518 (PMC4856970; doi:10.1093/nar/gkv1518)
Supplement: SUPPLEMENTARY DATA [file supp_gkv1518_nar-02692-z-2015-File007.pdf]

SUPPLEMENTARY DATA

CONBIND ALGORITHM

The pseudo code of the MSA algorithm described in the main text is shown below.

Algorithm 1 *Given a list of sequences (sequencesList) and a list of motifs families (motifsList), the following algorithm produces an aligned list of sequences using the motifs information to optimize the alignment.*

```
symbolsList = emptyList()
ForEach sequence in sequencesList
        ForEach motif in motifsList
                subsequencesList = searchForMotifs(sequence, getPatterns(motif))
                ForEach subsequence in subsequencesList
                        ForEach base in subsequence
                                symbol = getSymbolForBase(base, getFamily(motif))
                                symbolsList.add(symbol)
                                replaceLetter(subsequence, base, symbol)
                        EndFor
                EndFor
        EndFor
EndFor
extendedMatrix = getExtendedMatrix(symbolsList)
alignedSequences = performMSA(sequencesList, extendedMatrix)
ForEach sequence in alignedSequences
        ForEach symbol in symbolsList
                base = getBaseForSymbol(symbol)
                replaceLetter(sequence, symbol, base)
        EndFor
EndFor
Return alignedSequences
```

WEIGHTS ESTIMATION

The performance of our motif-aware alignment method depends on two parameters; the motif match weight (MMW) and the motif mismatch weight (MSW), as described in the method section of the main text. These two parameters are responsible for the trade-off between overall alignment score and the number of aligned TFBSs. Intuitively, the heavier the MMW and the MSW, the more TFBSs will be aligned on the expense of the overall alignment score. Furthermore, in case of extremely heavy weights, TFBSs that lay far apart on their respective

regulatory regions, can be forcefully pulled together in the alignment, artificially aligning unrelated TFBSs. Contrarily, the weaker the MMW and the MSW are, the more likely it will be for the algorithm to discard the information about motifs and optimize the alignments in the traditional way (i.e. maximising the alignment score). An optimal motif-aware alignment method should produce alignments with a minimal change in alignment score and, at the same time, be able to align all (and only) functional TFBSs. These two objectives, however, are clearly discordant. We can imagine the difference in alignment score as the Cost that we need to pay for a certain gain in Effectiveness, i.e. the number of functional TFBSs correctly aligned.

In order to find the best parameter values and assessing the quality of the produced alignment compared with the efficiency in identifying conserved TFBSs we trained our method using a set of regulatory regions for which the functional TFBSs where previously experimentally validated by the Göttgens Lab in murine cell lines (personal communication). This training set includes 14 regulatory regions (Erg+75, Erg+65, Scl+40, Cx3cr1 promoter, Gfi1b+16 Meis1+48, Gfi1b+17, Pim1+10, Lmo2-70, Scl+19, Lyl1+2, Fli1-15, Gata2-3, PU.1-14) for a total of 114 experimentally validated TFBSs belonging to six motif families (i.e. ETS, GATA, EBOX, GFI1, MEIS, and RUNT). These 14 mouse regions were aligned to seven different organisms (i.e. *Homo sapiens*, *Bos taurus*, *Canis lupus familiaris*, *Loxodonta africana*, *Monodelphis domestica*, *Sarcophilus harrisii*, and *Ornithorhynchus anatinus*) using ConBind. We performed an exhaustive parameter sweep running ConBind with different MMW and MSW pairs, such that $1 \leq MMW \leq 50$ and $0 \leq MSW \leq MMW$ (notice that assigning a heavier weight to a mismatch would not be a sensible option), for a total of 1325 runs. The sum-of-pairs score as defined by Thompson *et al.* (1999) was used to assess the overall quality of the produced alignments. For each ConBind run (with a specific MMW and MSW pair) two values were computed for each region *R*. The cost

$$Cost_R\left(A_R^{MMW,MSW}\right) = 1 - Sum - of - pairs\ Score(A_R^{MMW,MSW}),$$

where $A_R^{MMW,MSW}$ is the alignment of the region *R* produced by ConBind using the weight pair MMW,MSW. The columns corresponding to TFBSs were excluded in the Cost calculation. Notice that a perfect alignment yields a sum-of-pairs score of 1. For a perfect alignment *A* we expect $Cost_R\left(A_R^{MMW,MSW}\right)$ to tend to 0. The second value we computed is the effectiveness

$$\text{Effectiveness}_R\left(A_R^{MMW,MSW}\right) = \frac{\sum_{T_R^i}^{T_R} \frac{n(T_R^i)}{H}}{T_R}$$

where T_R is the total number of experimentally validated TFBS on the mouse region *R*. T_R^i is the *i*th experimentally validated TFBS on the mouse sequence, *H* the number of sequences used in the alignment and $n(T_R^i)$ is the number of organism (including mouse) in which T_R^i is aligned in the same position. Notice that for every i, $n(T_R^i)/H$ should approach 1, since all T_R have been experimentally validated and should be conserved in *H* sequences. Therefore, we expect $\text{Effectiveness}_R\left(A_R^{MMW,MSW}\right)$ to tend to 1 for an alignment A that shows highly conserved T_R. Supplementary Figure 1 shows the mean Cost and Effectiveness (over all 14 regions) computed

for every MMW, MSW combination. Notably, every weight combination shows an improvement in terms of efficiency over ClustalW2 alignments of the same regions.

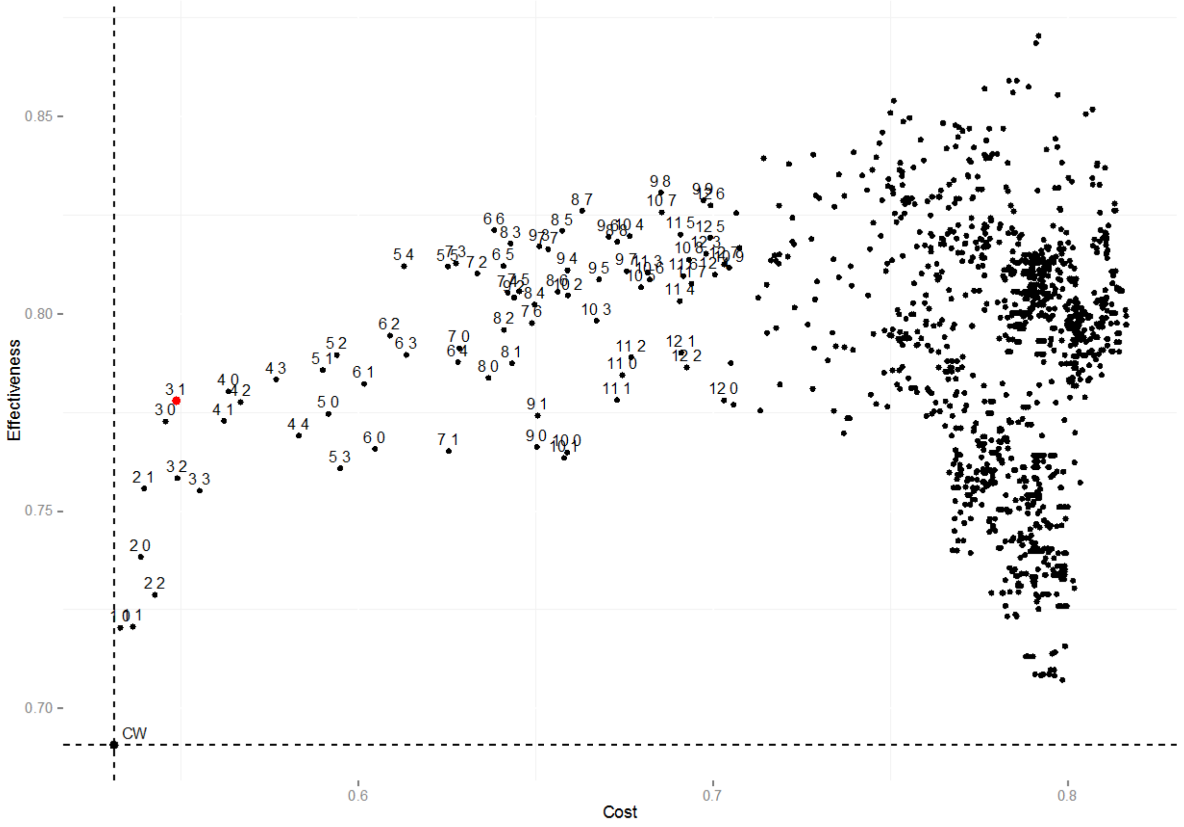

Supplementary Figure 1 *Mean cost and effectiveness of 14 benchmark regions for every MMW, MSW combination. Each dot corresponds to a weight pair, where the number on the left is the MMW and the MSW is on the right. The point labelled CW shows the cost and effectiveness computed using ClustalW2. The dot in red shows the weight pair with the best cost-effectiveness ratio after cross-validation.*

To reduce overtraining we computed cost and effectiveness using different subsets of the 14 regions. Specifically, we used every possible subset using 100%, 90% and 80% of the 14 regions, for a total of 379 subsets. For each subset we chose the MMW-MSW pair with the best cost-effectiveness ratio. For roughly 64% of the subsets the best weight pair had a MMW=3 and MSW=1. Supplementary Figure 2 shows the best weight pairs for the entire cross-validation study.

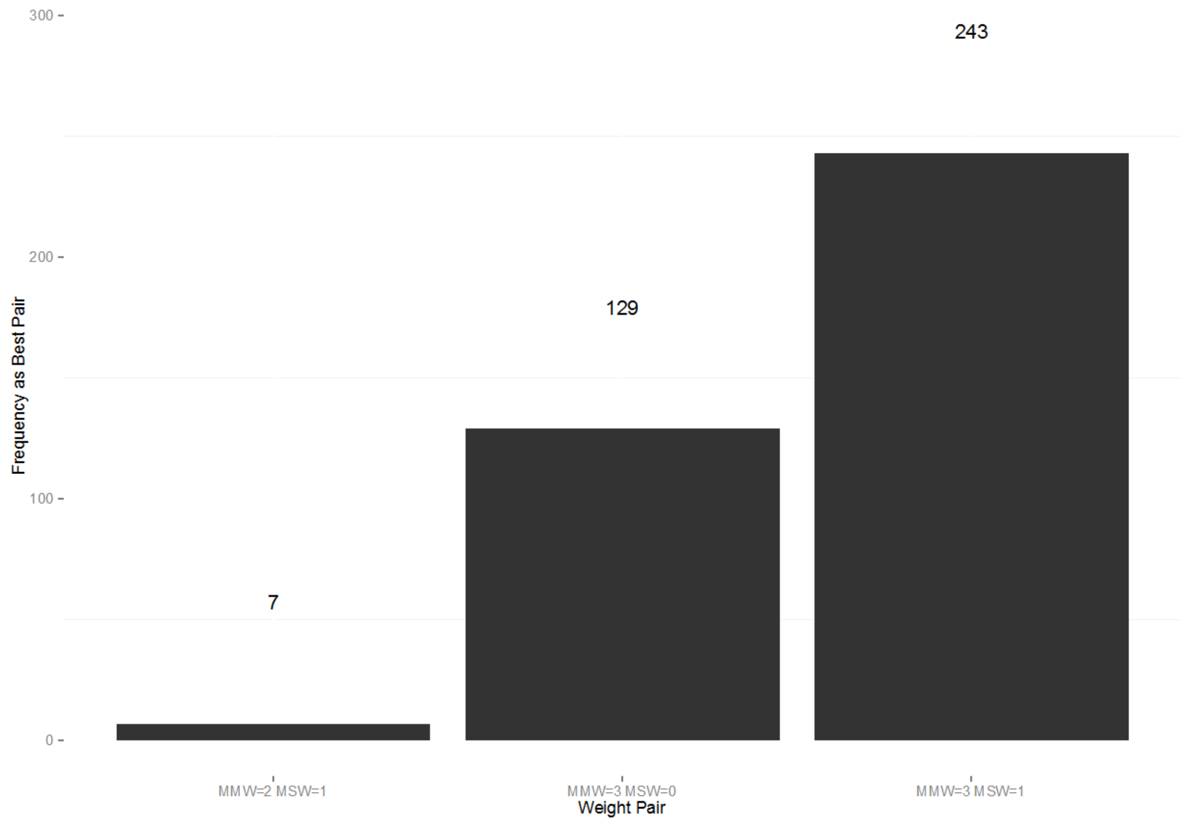

Supplementary Figure 2 *Only three weight pairs (over the total of 1325 tested) attained the best cost-efficiency ratio in at least one of the cross-validation subsets. Particularly, the pair with MMW=3 and MSW=1 is the one with the best cost-efficiency ratio for roughly 64% of the cross-validation subsets.*

Using the weight pair selected by cross-validation (i.e. MMW=3 and MSW=1) we compared the alignment of experimentally validated TFBSs between ConBind and ClustalW2, as shown in Supplementary Figure 3.

PRALINE AND PROGRESSIVE MULTIPLE SEQUENCE ALIGNMENT

The progressive multiple alignment step was performed with a flexible sequence alignment program, a reimplementation of the available PRALINE MSA toolbox (Heringa, 1999). This tool was developed in-house and thus has support for required features such as the use of custom symbol alphabets and weight matrices during the alignment process. In order to reduce the number of parameters, we used default settings and implemented a minimal tree-guided progressive alignment strategy. A reasonable default was chosen for the linkage method during the hierarchical clustering (UPGMA).

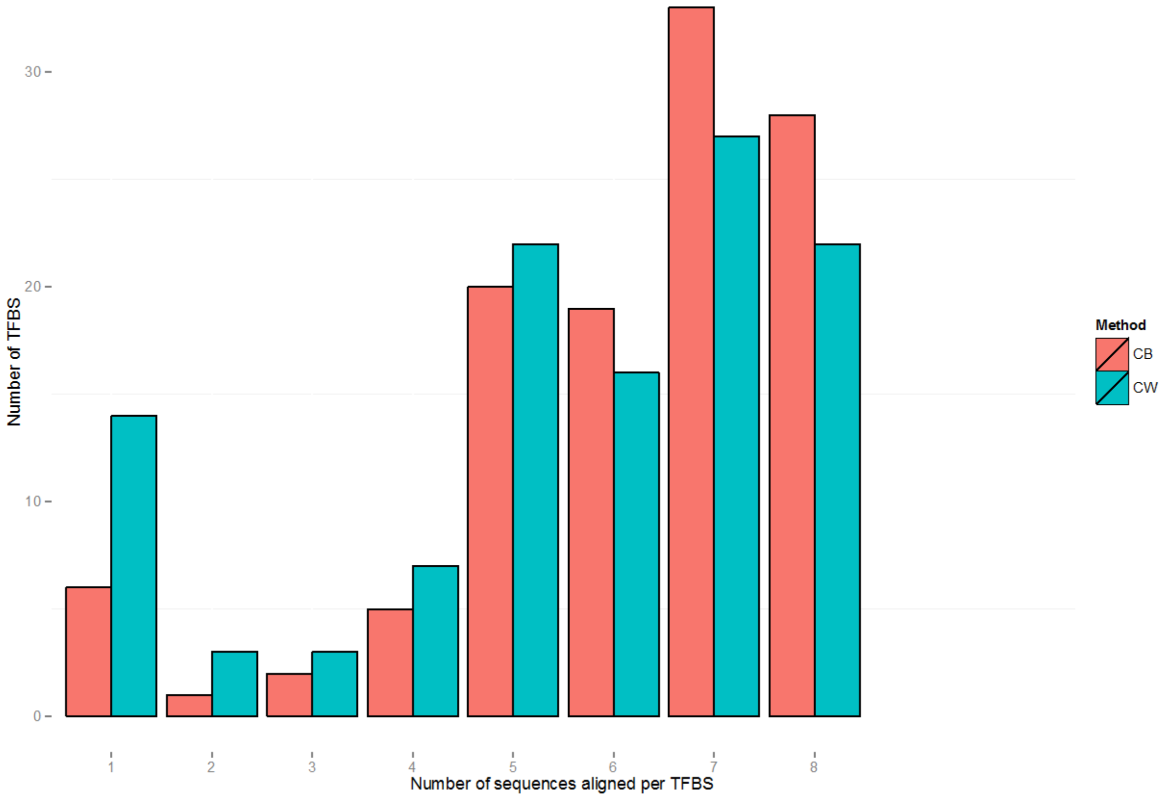

Supplementary Figure 3 *For each experimentally validated TFBS we counted the number of species in which the TFBS was aligned in the same position. ConBind (CB) can detect a higher conservation signal compared to ClustalW2 (CW), indicating that ConBind aligns more TFBSs.*

GENERATING THE GFI1B+13 CONSTRUCTS

<u>Gfi1b+13_wt (chr2:28,457,606-28,458,256; mm9)</u>

```
CAGGTGCTAGATCCCGTCATTTGGGACCAATACCTAGTTGTCCCTAGTAAATTTATGTCTACAGGGACCTGGAAC
CTTGGCAGTTAGAACAGAATTTCCTAGGTAGAGCAGGGCCCTGCCTTAGGAACTGAGATCTGGACAGTGGACACT
TGACTCTTCCTAGGACACACAGAATTAGTTCTGGGAAGATGCCACCCCAGTGGCCCCCATAGATCTAGCTGGGGT
TGAGCCCTGCCAGGAGCCAGGCTGGCCCTGTGCTTCTCGGGAACCATGAGTGCAGAAAGGCAACTGGAGGGAAAT
CTGAGGCAGGACACGGTCAGACCTAGCCATGCTCAGGTTTGGCCTGATAAGTTTATCAGCCTGTGCCGGCTGCCA
ACTGTCAGCATGGAGCCTGGCAGGGGGCTGGGTGGGAGGACACTCCTGGGTCGATAGCGCCTTCCAAGTGTTATC
AGGGGCACCGTGGCCCAGAGCGCGGGAAACGGGTGAAACAGGAGAGAAAGAGACTTCCAACCACTTTACCCAAAG
AAAAGCACTGGGAGGGGAACCGAGGCCTCAGTGTTCCTGGACCCTGACCTGCTGTGAAACCAGCAGTCACAGCTG
AGTCCCAGGGAGGCACAGGCTGAGGACCCTGCCACAGACATCCAGAGGGAA
```

Gfi1b+13 mutants for Gfi (yellow) and Ebox (blue) were generated using standard recombinant DNA techniques using the primers listed below.

Gfi1b13_Gfimut_Fw	ggaattctgaggcaggacacggtcagacc
Gfi1b13_Gfimut_Rv	agaattccctccagttgcctttctgc

Gfi1b13_Eboxmut_Fw ttcctgcagttatcaggggcaccgtggc

Gfi1b13_Eboxmut_Rv taactgcaggaaggcgctatcgacccagg

The Gata (green) and Ets (purple/red) mutants were generated by GeneArt® Gene Synthesis (Gata and Ets1-2) or GeneArt® Strings™ (Ets3-5) from Life Technologies. The whole Gfi1b+13 enhancer fragment with the relevant point mutations was ordered and subsequently cloned into pGL2 promoter vector from Promega.

Gfi1b+13_Gata (generated by GeneArt® Gene Synthesis from Life Technologies):
1st GATA-GGTA, 2nd TATC-GATC, 3rd GATA-GGTA, 4th TATC-GATC

Gfi1b+13_Ets1-2 (generated by GeneArt® Gene Synthesis from Life Technologies):
1st: GAGGGAA – AAGCTTA 2nd: GCCTTCC – GCTAGCC

Gfi1b+13_Ets3-5 (generated by GeneArt® Strings™ from Life Technologies):
3rd: TTCC – TTGC 4th: GGGAAC – GACGTC 5th: TTCC - TACC

Supplementary Figure 4 *ConBind identifies conserved TFBSs within the Lmo2-75 enhancer.* ***A.*** *The previously described, hematopoietic active regulatory region for the Lmo2 gene (called Lmo2-75, (1)) is 3.5 kb long and is comprised of two sub-regions (region 1 marked in red, region two marked in yellow) that are bound by several TFs in the hematopoietic progenitor cell line HPC7.* ***B.*** *Luciferase reporter assays in stably transfected 416b cells, a myeloid progenitor cell line, reveal that both sub-regions of this enhancer are transcriptionally active on their own. Shown is the relative luciferase activity of the wild-type (wt) enhancer compared to an empty control vector.* ***C.*** *ConBind's algorithm results in aligning two ETS motifs, one Ebox and one Gata motif within sub-region 1, matching the TFs binding to this part of the enhancer. ConBind is able to find additional ETS and GATA motifs (six and four in total, respectively) within sub-region 2 of the Lmo2-75 enhancer.* ***D.*** *Manual identification of TFBSs within the two TF-bound sub-regions of the Lmo2-75 enhancer was performed as described in Figure 4A. Despite binding of Erg, Gata2, Lyl1, Runx1 and Scl (see A), no conserved TFBS could be observed within sub-region 1. In contrast four conserved ETS motifs, two conserved GATA sites and three conserved GFI1 motifs are located within sub-region 2 where binding of the ETS factors Erg, Fli1 and PU.1 as well as Gata2 can be seen (see A).*

1. Landry, J.R., Bonadies, N., Kinston, S., Knezevic, K., Wilson, N.K., Oram, S.H., Janes, M., Piltz, S., Hammett, M., Carter, J. *et al.* (2009) Expression of the leukemia oncogene Lmo2 is controlled by an array of tissue-specific elements dispersed over 100 kb and bound by Tal1/Lmo2, Ets, and Gata factors. *Blood*, **113**, 5783-5792.

A

Lmo2-75: chr2:103733174-103736735

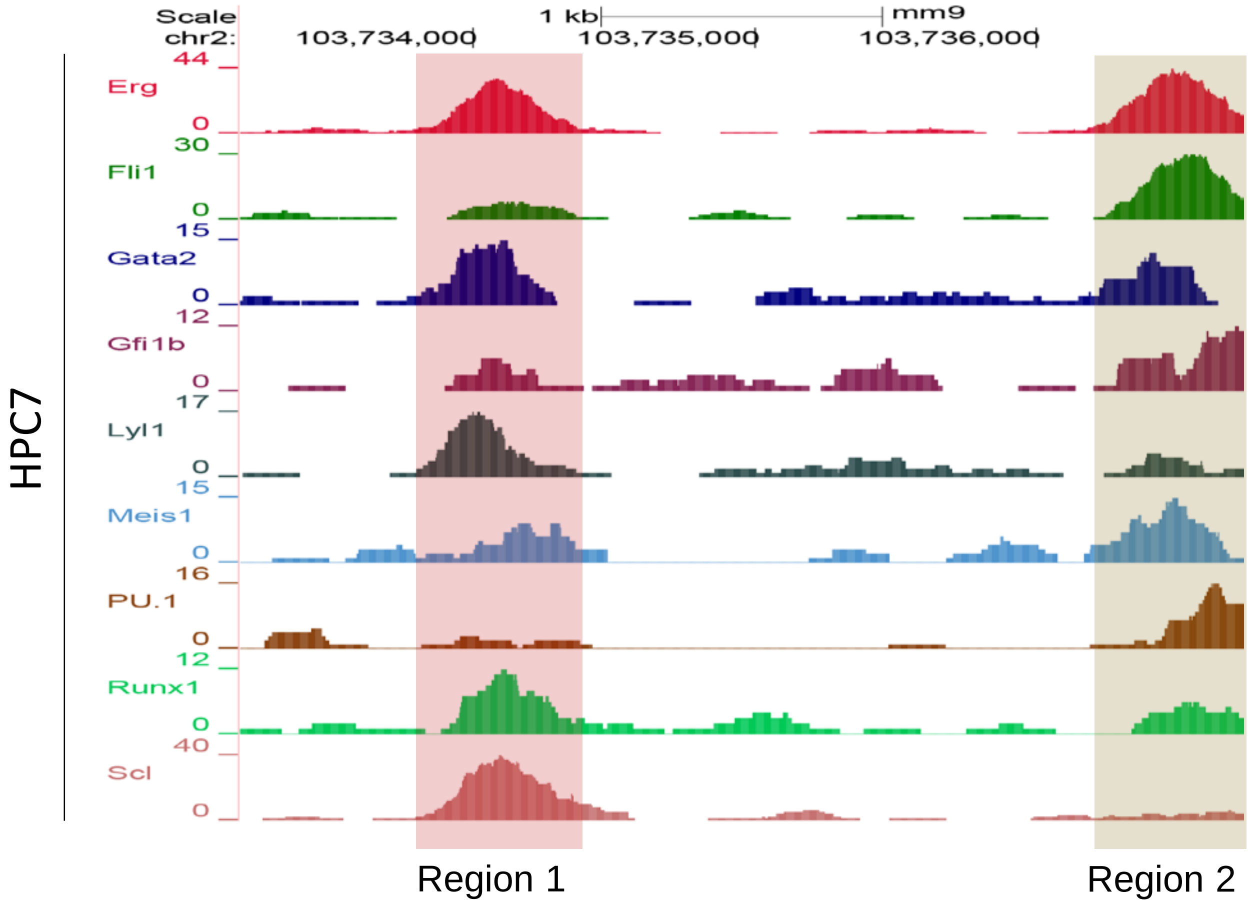

B

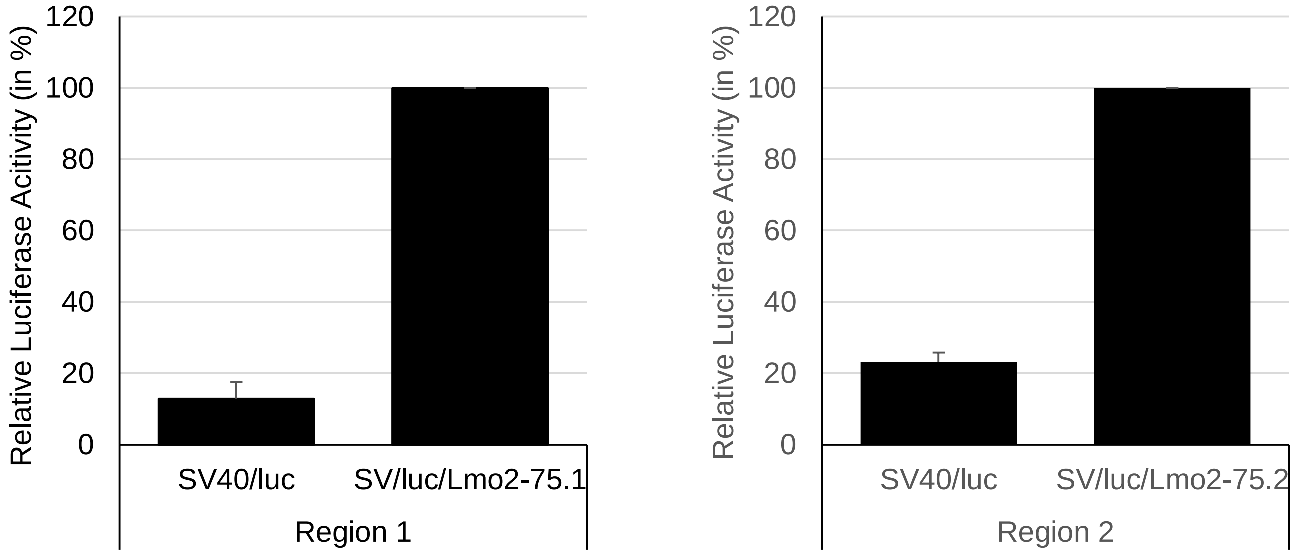

C

http://www.conbind.org/view/f6bc8728-e6ae-405b-b430-8e292016a3dc - 1

Legend

EBOX ETS GATA GFI1 MEIS RUNT

Alignment Quality: Low High

Lmo2-75

chr2:103733174-103736735

```
10090 (house mouse)                               CTTCTTGGGGCTTTCTTGGATCCCTCCAGAACTTTCCTCTTTTTGATTTTTTTTATAA
9615 (dog) e-value: 5e-75                         ----------------------------------------------------------
13616 (gray short-tailed opossum) e-value: 1e-46  ----------------------------------------------------------
9606 (human) e-value: 3e-111                      ----------------------------------------------------------
9258 (platypus) e-value: 8e-40                    ---------GAGCAGAGGGA-------A-AGGGGGAAAAAGAGGGTTTAGCTGCGGAG

10090 (house mouse)                               AGGCAAAAATTTAATTGGGAATAACTTACAGATGCAATAAGCATCCATTATTATTATG
9615 (dog) e-value: 5e-75                         -----------------GAGATGCATGCGTGAGGGGGACAGGTTCC------A--ATG
13616 (gray short-tailed opossum) e-value: 1e-46  ----------------------------------------------------------
9606 (human) e-value: 3e-111                      ----------------------------------------------------------
9258 (platypus) e-value: 8e-40                    AGGTAAAAGGGGGGTGGCAGAGGGAGTAGAG-GGAGAAGAGGAGCTCAGTCTGGGAAG

10090 (house mouse)                               GCATCATGCAGGCAGACTTGATGTTTGAAGAGCCTCCAGCCTTGATTCAAAGACAGCC
9615 (dog) e-value: 5e-75                         AGAGTAGCCAGTCTG--GTGAAGGACT-CAAGCTTT----CTGGGTGGAGACGTCTCA
13616 (gray short-tailed opossum) e-value: 1e-46  ----------------------------------------------------------
9606 (human) e-value: 3e-111                      ----------------------------------------------------------
9258 (platypus) e-value: 8e-40                    GCCTC-TT--GGA------G----GAGGTGAGTTTTAAG-TAGGGTTTTGAAGA----

10090 (house mouse)                               AAGAGGATGATATCTTCTGCATTGGGTAGAGCTTGAGTATAGGACCTCAAAGCCCATT
9615 (dog) e-value: 5e-75                         GAAGGGAT-----------------GTGCCAGTTCTGTTCAAATATTTGAAG--CATT
13616 (gray short-tailed opossum) e-value: 1e-46  ----------------------------------------------------------
9606 (human) e-value: 3e-111                      ----------------------------------------------------------
9258 (platypus) e-value: 8e-40                    --GGGGAAGAGA----ATCAGTTTGGTGGAGG-----T----GAGGAGGGAGGGCGTT

10090 (house mouse)                               CC---CACAGTGACTCACTTCTTTCAACAG--GCCA-C-------ACC-TACTCTAAC
9615 (dog) e-value: 5e-75                         TC-------------------------------------------------------C
13616 (gray short-tailed opossum) e-value: 1e-46  ----------------------------------------------------------
9606 (human) e-value: 3e-111                      ----------------------------------------------------------
9258 (platypus) e-value: 8e-40                    CCAGGACCGCGGGAGGACGTGGTCCAGGGGTCGACGGCGGGATAGGCGAGACCGAGTG

10090 (house mouse)                               AAGGCC---ATATATACCTCCTAATAGTGCCACTTTTCCACTTCCCCTGGGCCAAGTA
9615 (dog) e-value: 5e-75                         -A---C-----ATAGACCGCCACAGAG-ATCGGACTTAAATTTCC-------TACGG-
13616 (gray short-tailed opossum) e-value: 1e-46  ----------------------------------------------------------
9606 (human) e-value: 3e-111                      ----------------------------------------------------------
9258 (platypus) e-value: 8e-40                    ACGGTGAGGAGGTGGGCGGCAGAGGAGCGGAGCGTGCGGGGTGGGCGGTAGAAAGAGA

10090 (house mouse)                               TATTCAAGCCACCATAGCACCCCTCAAAGAGCTGGTTGCAGTTTGATTTTTATTAGTA
9615 (dog) e-value: 5e-75                         --TTCTGGAGAGCACAGCAAGAACCAAA-CGATGGCTCCA------------------
13616 (gray short-tailed opossum) e-value: 1e-46  ----------------------------------------------------------
9606 (human) e-value: 3e-111                      ----------------------------------------------------------
9258 (platypus) e-value: 8e-40                    GAAGGGAGGAGAGGTAGGAAGGGGCAAGGTGATGGAGAGCCTTGAAGCCTAGAGTGAG

10090 (house mouse)                               CTGATTTGCCTTTTGTTTTCACTTTGA--GTACACTCTGAACTT-ATT--------GA
9615 (dog) e-value: 5e-75                         --------------G---------AGA--CACAACCTTGGAC-----T--------CA
13616 (gray short-tailed opossum) e-value: 1e-46  ----------------------------------------------------------
9606 (human) e-value: 3e-111                      ----------------------------------------------------------
9258 (platypus) e-value: 8e-40                    GAGTTTTTGTTTGGAGCGGAGGTTGATAGGCAACCACTGGAGTTGTTTAAGAAGGGGA

10090 (house mouse)                               GTGACACTGACCTCCAGTCTTCTAGTGATCTTAAACTTGGTCAAGGGAGAAAT-CT--
9615 (dog) e-value: 5e-75                         ATGACA----GCAAGAAAGGTCCATCAACCAGAACTTTCTGAAAAGGAAATGA-GC--
13616 (gray short-tailed opossum) e-value: 1e-46  ----------------------------------------------------------
9606 (human) e-value: 3e-111                      ----------------------------------------------------------
9258 (platypus) e-value: 8e-40                    GTGACA-------------------TGCCCAGATCATTTCTGCAGGAAGATGAGCCGG
```

```
10090 (house mouse)                                 -C-TCTGGTTTTGCTTGCTACTATAGTTCCATGCCAACTACAGTGCTTAGCACACAGT
9615 (dog) e-value: 5e-75                           -C-ACCTTTCTGGAAGTGTTCAAGCCTGGCCTG---GGTATGGTCGTTTATTCCATTC
13616 (gray short-tailed opossum) e-value: 1e-46   ----------------------------------------------------------
9606 (human) e-value: 3e-111                        ----------------------------------------------------------
9258 (platypus) e-value: 8e-40                      GCAGCAGAGTGAAGAATAGACTGGAGCGGGGCGAGAGAGGAGGAAGGGAGGTCAAAGA
```

```
10090 (house mouse)                                 AGGGACTCATAAGTAAGTTACCAATAGATA-------AGTTCATGGCTGGCTGGCTGA
9615 (dog) e-value: 5e-75                           A--G---------ATATGTAATGAGGGATG-------TTCCCTAGGCCTGCTGTTTGT
13616 (gray short-tailed opossum) e-value: 1e-46   ----------------------------------------------------------
9606 (human) e-value: 3e-111                        ----------------------------------------------------------
9258 (platypus) e-value: 8e-40                      GAAGGCTGACACAGTAGTCTCTCCGGGATATAACGAGAGCCCGTAGCAGTAAGGTAGC
```

```
10090 (house mouse)                                 ATAAATGAATGAATGGATATTTTATAAGTGGTCAAGGATGCAAAACATCAAAGTAACA
9615 (dog) e-value: 5e-75                           GAGAAAG-A--GCTGGAT--GCTGGGAGTAGGGGAGAGAGAGAATCTAATATGACAAA
13616 (gray short-tailed opossum) e-value: 1e-46   -------ATCTCAGACAATTCTTAGCTGTGTGACCCTGGGCAAATC---------ACT
9606 (human) e-value: 3e-111                        ----------------------------------------------------------
9258 (platypus) e-value: 8e-40                      CGTTTGGGTGGAGAGGA-A-------A----GGGCGGAT-------------------
```

```
10090 (house mouse)                                 TGCTTAAAGGTACACCATAGCAAAGCTGGTTCTATATGACAAC-GACCAA-G--CGGA
9615 (dog) e-value: 5e-75                           CAATTAAAAG----CCATTCAT-TAGTAGCTCAATAT-------G--TGGGTTTCCAG
13616 (gray short-tailed opossum) e-value: 1e-46   TACTTCCCATTGC---CTAGCCCTTACTGCTC--------TTCTGTCTTTGATTCCAA
9606 (human) e-value: 3e-111                        ----------TGCGTGATAGTCATGACTGTTCAATATGACTTCGGACCAAGGAATTTG
9258 (platypus) e-value: 8e-40                      ---------CTTGGCGATA--TTGTAGAGGTGAAACTGACA-----------------
```

```
10090 (house mouse)                                 GGCTGTATTATTACATTAGGTAGAACTCAAAGCCAAAGTCAACCATTCCCTTTGAACT
9615 (dog) e-value: 5e-75                           CACTTACATGC-ACC-AAGGACCGAACTAACTACTTTGTGGGCATGACCACTTAATCC
13616 (gray short-tailed opossum) e-value: 1e-46   GACAG-AA----------GGAAAGAAAGGAAG--AAAGAAAGAAAAAGAAAAAAAGAG
9606 (human) e-value: 3e-111                        GACTGAAATGCTCATTAGAGAGCAACTAAAAGCCAAAGTCAGCTATTCCCTTTGAACT
9258 (platypus) e-value: 8e-40                      ----------GGTCTTGGTAACAGATCGGATGTGTGGGGTGAACGAGAGAGACGAGTC
```

```
10090 (house mouse)                                 CAGTGTGATA-----GAGTA--G-----AGTTGAGCCATTATCTTCTGAGGCAGCTAA
9615 (dog) e-value: 5e-75                           T--CATGACA-----ATCTTGGGCATTAAGTCCTACAATTATC------------ACC
13616 (gray short-tailed opossum) e-value: 1e-46   ---GAAGA-AA-----TAAAAAGAAATAAGG-GAGGAAA-------------------
9606 (human) e-value: 3e-111                        AGGTATGACA-----AAGTT--G-----GGACTGGATATTATCTTCTGAAGCAGCTAA
9258 (platypus) e-value: 8e-40                      AAGGATGACACCGAGATTGCGGGCCTGAGAGACGGGAAGGATGGTCGTGCCATCCACG
```

```
10090 (house mouse)                                 CTGTTACAGGAGAAGGAGGGCTGAGGAGGGGGAAGGGGGGTGGCTCAGGCCCAAATTA
9615 (dog) e-value: 5e-75                           CTGTTAGA-G----------------ATCGGGAGACTGACACTGAGAGGAGTGAAATA
13616 (gray short-tailed opossum) e-value: 1e-46   --GAGAGA-AAGAAGGAAAGAGAATGAGAAAGAGAGAAAGAAAGA-AGGAAGAAAGAA
9606 (human) e-value: 3e-111                        CTGTTAC------ATGA----AGACAAGGGAG-TGGGAGGTCTCCTGGGCCCAAATCA
9258 (platypus) e-value: 8e-40                      GTGATAGAGAAGTCTGGGAGAGGAC-CGGGTTTGGGAGGGAAGATGAGGAGCTCAGTC
```

```
10090 (house mouse)                                 AGTGCCTCAGAGGCTGTAGTAT-ACTCTCCCAGATCCCAGATGCTCACACAACCTGGC
9615 (dog) e-value: 5e-75                           ATTTGCCCCAA-------G--------TCACACAGCGAGGAAG--TACAGAGCTGGGA
13616 (gray short-tailed opossum) e-value: 1e-46   AGAAANNNNNNNNNNNNNNNNNNNNNNNNNNNNNNNNNNNNNNNNNNNNNNNNNNNNN
9606 (human) e-value: 3e-111                        AATGCATCAAAGACCATAGTGTCCCTTCCCCAGATCCCAGATGTTCACACAACATGGC
9258 (platypus) e-value: 8e-40                      TTGCTCATGTTGAGTTTTAGGTGGCGGGCCAACATC-CAGGTG---------------
```

```
10090 (house mouse)                                 CTGAGAGATTAGAGCTGGTCTTCCTGTGGCT--C-----C--C--T---GCTGTTATC
9615 (dog) e-value: 5e-75                           CT--CA-AAT------------CC-------------T--AACCACCAAGCTATTA--
13616 (gray short-tailed opossum) e-value: 1e-46   NNNNNNNNNNNNNNNNNNNNNNNNNNNNNNNNNNNNNNNNNNNNNNNNNNNNNNNNNN
9606 (human) e-value: 3e-111                        CAGAGAGATTAGTACTGGTCTTCCTGTGGCTCCCTGATGCTGCTATCCAGCTGTTATC
9258 (platypus) e-value: 8e-40                      ---------------GAGACATCC-------------T-----------G-------G
```

```
10090 (house mouse)                                 TGACCCAACCTGCTGCCTGAC--ATTGCAGGAGGCTGAGTCTGGGCCCCACCTCAAAG
9615 (dog) e-value: 5e-75                           ---CCCATTCTGCT-AC-AGC--ACC-CT----GC-TCCCCTCTGCCTCCCCTC----
13616 (gray short-tailed opossum) e-value: 1e-46   NNNNNNNNNNNNNNNNNNNNNNNNNNNNNNNNNNNNNNNNNNNNNNNNNNNNNNNNNN
9606 (human) e-value: 3e-111                        TGGCCCAACCTGCTGCCAGGC--CTTG-GGGAGGCTGAGCCACAGCCCTGCCTCAGAG
9258 (platypus) e-value: 8e-40                      AGGCAGGAGGAGATGCGAGCCTGAAGGGAGGGGGAGAGGACAGGGACGGAGATGTAGA
```

```
10090 (house mouse)                                 GGGCCCT-ACATCTTCTCAGTAAAACTACTTCCAGCTCATGCGTGGGGTTGGAGTGAA
9615 (dog) e-value: 5e-75                           --TACCTCCCATTCCTTCCTATTAGAGCATAAACGCTATTA--GACCATTAAAAGAAA
13616 (gray short-tailed opossum) e-value: 1e-46   NNNNNNNNNNNNNNNNNNNNNNNNNNNNNNNNNNNNNNNNNNNNNNNNNNNNNNNNNN
```

9606 (human) e-value: 3e-111 GGGCCCTGTCATCTTTCCATTAAAGCTACCTCCA----TTACTTCTAGTGACGGAAAT
9258 (platypus) e-value: 8e-40 TCTGCGTGTCATCTGCATAGAGATGGTAGTCAAAG------C----CATGAGAGCGAA

10090 (house mouse) AGTGTCCCACTGACGATAGAGGAGGAGAGGATTGGTCTCTAGAATGACCTCCTGTCTG
9615 (dog) e-value: 5e-75 TTTCTCCCA------AGAAAGTTCGTCAGAATGGGCCCCAACACAGAACAGCAACTAA
13616 (gray short-tailed opossum) e-value: 1e-46 NNNNNNNNNNNNNNNNNNNNNNNNNNNNNNNNNNNNNNNNNNNNNNNNNNNNNNNNNNNN
9606 (human) e-value: 3e-111 GTTCTACCAGCTACAGGGCATTGGGGTGTGCTTAGTCTCCAGAACCACCTCTCATTAA
9258 (platypus) e-value: 8e-40 TGAGT-------TC-------ACCGAGGGAGTGAGTGTAAATGGAGAACAGAAGAGGG

10090 (house mouse) TCACCTAACCCTCATGGTTTAGGTGCCACA--GG-AGTGGA--------G-------A
9615 (dog) e-value: 5e-75 CAACAGAAAACGCCCAAATTGGGGGAAATTACTACATTTGAGTACAATTTGCAAATCA
13616 (gray short-tailed opossum) e-value: 1e-46 NNNNNNNNNNNNNNNNNNNNNNNNNNNNNNNNNNNNNNNNNNNNNNNNNNNNNNNNNNNN
9606 (human) e-value: 3e-111 TCAGCCACCTCCAACCATTTTGGTGCCATAGGGGCAGAGATTCACCTCCGTAACAAAA
9258 (platypus) e-value: 8e-40 CCA-AGAACTGACCCTTGCTCGTTGCCCGTACCCCCTCTAA-TAA-TAT-T---AATA

10090 (house mouse) -CTTAGAAATGTGCAACGTATTCCCTGCTAACTGGGTGAGCCTTCTGATGTCTTAGAT
9615 (dog) e-value: 5e-75 TCT-TGGGA----GAGAGTCTTCCC----------------------------------
13616 (gray short-tailed opossum) e-value: 1e-46 NNNNNNNNNNNNNNNNNNNNNNNNNNNNNNNNNNNNNNNNNNNNNNNNNNNNNNNNNNNN
9606 (human) e-value: 3e-111 TATATGAAACTTGGATGGTGTTTCCTGTTAAACGAATGAGTCTTCTGATTTCTTTGAT
9258 (platypus) e-value: 8e-40 ATGTTGGTATTTGTTAAGCATTTACTATGTGCAGAG-CACTGTTCTAAGCCCTGGGGT

10090 (house mouse) CTCAGCGTCCCGTGAAATTGAGTGGGGATA---------A-CTTCCACAGTCTACGGC
9615 (dog) e-value: 5e-75 ------------GCAAAGTGAGAGCCAATTTTAAAACACCCACATCCTC-----TG--
13616 (gray short-tailed opossum) e-value: 1e-46 NNNNNNNNNNNNNNNNNNNNNNNNNNNNNNNNNNNNNNNNNNNNNNNNNNNNNNNNNNNN
9606 (human) e-value: 3e-111 CTCACCCTCCTACCAGACAGA-AATTGATAAATGGGGACATGTTCCAATGAGAGTGAC
9258 (platypus) e-value: 8e-40 -------------------------AGATA----------CAGGGTAATCAAGTTGTC

10090 (house mouse) ACACCTCA-GGAAAGCGCTCTCAA-CTAT-TCCAAGTGAAC-AGCT---AAAGGGTGT
9615 (dog) e-value: 5e-75 --GCCTCACTCCCTCCTC-CCCAAAG---GTTCATCTGGCCTAGCTCGGCAAAAA--T
13616 (gray short-tailed opossum) e-value: 1e-46 NNNNNNNNNNNNNNNNNNNNNNNNNNNNNNNNNNNNNNNNNNNNNNNNNNNNNNNNNNNN
9606 (human) e-value: 3e-111 CAGCCTCATGGACTGGACTCACAAGCATTCTGGATGGAGAC-ATCTCGGAAGGAATAT
9258 (platypus) e-value: 8e-40 CCACATCAGACTCA-CAGTTAGTCCCATTTTACAGATGAGGTAACT------------

10090 (house mouse) GCCAATTCTTTTAAGGGATTTGAAGGATTTCCATGTAGAACAAAAGCCAGA-C-GAGT
9615 (dog) e-value: 5e-75 -----CCCTTTGCCTTCATTTGCATTG-TT---CTTGGGAGGAG-GAAAG---TGAGC
13616 (gray short-tailed opossum) e-value: 1e-46 NNNNNNNNNNNNNNNNNNNNNNNNNNNNNNNNNNNNNNNNNNNNNNNNNNNNNNNNNNNN
9606 (human) e-value: 3e-111 ACCAACTCTTTTGTAATATTTGATGAGTTTCCACACAGAAGCAGTGTTAGACCTAAAT
9258 (platypus) e-value: 8e-40 -------------GAGGCACAGAGAAGTTGTGACTTGCCCCCAGTCACACAGCTGACA

10090 (house mouse) TGTCTGTGATTCTGGGGCTCTCAGCAAGCAGTAAGTGATGATTACAGAGTGTTTGCCT
9615 (dog) e-value: 5e-75 GAGCCCTG---CAGTCACTCCCCCTGAGAAGACAGTCA-GAATCAC-A-------CCT
13616 (gray short-tailed opossum) e-value: 1e-46 NNNNNNNNNNNNNNNNNNNNNNNNNNNNNNNNNNNNNNNNNNNNNNNNNNNNNNNNNNNN
9606 (human) e-value: 3e-111 TTCCTATGGTTCTGGAGAGCACAGCAAAAACTAAATGATGGCTCCAGAGCAACAGCAT
9258 (platypus) e-value: 8e-40 AGTGGCAGA-GCCGGGGTTCAAACCCA--TGAC--CTCTGACTCCCAAGCCCGTGC-T

10090 (house mouse) TTGACAAAAGTGAAAAGGAGAAATGTCTGCTAACCATAGCTGTCTGAACACTCCCTCC
9615 (dog) e-value: 5e-75 TCAACTCAA-TCG---GCCGTGACTTCTTGGGA--ATTTGG--CTGAAGGGTCCCAAA
13616 (gray short-tailed opossum) e-value: 1e-46 NNNNNNNNNNNNNNNNNNNNNNNNNNNNNNNNNNNNNNNNNNNNNNNNNNNNNNNNNNNN
9606 (human) e-value: 3e-111 TTAATTCAAAT-AAAAGAAGAAATCTCTATCAACC--A-------GAATGATCTG---
9258 (platypus) e-value: 8e-40 CTT--TCAACTG------------------AGCCACGCTGCTTCTCTAGGCTGTAAGC

10090 (house mouse) CGACCCCCACTCCGAACCTTTCCTCCCCCCCCCCCCCGCCCCCCCGCCAGCTGAATAC
9615 (dog) e-value: 5e-75 CGAGTCAAACT---TAATTAGTCAACTTTTACCCCAGAGCATGTAGACACTTGA-TGC
13616 (gray short-tailed opossum) e-value: 1e-46 NNNNNNNNNNNNNNNNNNNNNNNNNNNNNNNNNNNNNNNNNNNNNNNNNNNNNNNNNNNN
9606 (human) e-value: 3e-111 -------------AA---------------------------AACGAAATGGACTAC
9258 (platypus) e-value: 8e-40 TCGTTGTGGGCAAGGAATGTGTCTA-TTTTT--CTATTTTACTCTCCCAAGTGA----

10090 (house mouse) CCAGCAGGGGATTGAGTTGCCTCACCCCATCTTTGGTGGAGTATGAGCTTGGCCTGAG
9615 (dog) e-value: 5e-75 TTTGTAAGAAAATGAG-T---GCACCATACCCGT---------TGACAAG-----AAA
13616 (gray short-tailed opossum) e-value: 1e-46 NNNNNNNNNNNNNNNNNNNNNNNNNNNNNNNNNNNNNNNNNNNNNNNNNNNNNNNNNNNN
9606 (human) e-value: 3e-111 CTAGTTGAGTAGTGA---G--TCTCCT-GTCC------AAGCCTTACCTGGGTATGAG
9258 (platypus) e-value: 8e-40 -TT--A--GTACAGTGCT-CTGCACACAGTAAGCTCTCAATAAATACGATTGACTGA-

```
10090 (house mouse)                                 CTGTGTGTTCTATTCTAG-GATATACATAAT---GGGTGCTGTGTGAGTCTGCTATTC
9615 (dog) e-value: 5e-75                           ATGTGGGTGCTGGTC-AC-CAAAAAGAAAATAGAAAGGGGGAAGAGTGGTGGTTATTT
13616 (gray short-tailed opossum) e-value: 1e-46   NNNNNNNNNNNNNNNNNNNNNNNNNNNNNNNNNNNNNNNNNNNNNNNNNNNNNNNNNN
9606 (human) e-value: 3e-111                       TTGTATATTTCATTCTAT-TACACATATAATAAGGGACGTTTCGTAGGTAGGCTATTC
9258 (platypus) e-value: 8e-40                     CTGACTGTAATATCATTCACACTCTCCTCTTAAATGGCACCCAGCGTGGAGTCTATTT

10090 (house mouse)                                 CAAAGGAAGGTCTTGACAC--AGGAGCAGGGAA---GGAT--GC---T--TAGCACAA
9615 (dog) e-value: 5e-75                           ATTTATAAAGACTTTATTTATTTATTTATTCATGAGAGATACGGAGAGGCAGAGACAC
13616 (gray short-tailed opossum) e-value: 1e-46   NNNNNNNNNNNNNNNNNNNNNNNNNNNNNNNNNNNNNNNNNNNNNNNNNNNNNNNNNN
9606 (human) e-value: 3e-111                       CTGAGGAAGGTCTGGACACTGAGAAGTAGGGAAAAGGGATACTCTAATACAAATAAAA
9258 (platypus) e-value: 8e-40                     TAATGTTC-----CCCTAAATATTAGGACCTTAGTTTTATTCTCACACCTAATTAAAC

10090 (house mouse)                                 CT-TAAAAACCATTTAATAGCTCAATATGTTGGCTCTGAGCA--TCTATGTTCACTGA
9615 (dog) e-value: 5e-75                           AG-GCCGAAGGAGAAGCAGGCTCCCTGTGGGGAACCTGA--------------TGTGG
13616 (gray short-tailed opossum) e-value: 1e-46   NNNNNNNNNNNNNNNNNNNNNNNNNNNNNNNNNNNNNNNNNNNNNNNNNNNNNNNNNN
9606 (human) e-value: 3e-111                       AT-TAAACATCA-TTAGTAGCTCCCTATGTGGGTAATGAGCACTTCCATGTAC-CAGC
9258 (platypus) e-value: 8e-40                     GTAGAAGTTCCAGCTCTCCTAGACCTCTATTTCCACTTACCTTTTCC----TCTCTGA

10090 (house mouse)                                 ACGCCATAGCCCTGTACAGAAGCC------T--------GATA---------------
9615 (dog) e-value: 5e-75                           GACTCGATCC------C--AGGATCACGCCTGAGCCAAAGACAAACACTCAACCACTG
13616 (gray short-tailed opossum) e-value: 1e-46   NNNNNNNNNNNNNNNNNNNNNNNNNNNNNNNNNNNNNNNNNNNNNNNNNNNNNNNNNN
9606 (human) e-value: 3e-111                       ACAACAATCCTGTGCAGTGAGTCCTATAATTACCACCATGATACAGGCAAGGAGACTG
9258 (platypus) e-value: 8e-40                     CTTCAATTCCCCT-----------------------------CATTTCTTGCAGGCAA

10090 (house mouse)                                 ----------------------------------------------------------
9615 (dog) e-value: 5e-75                           AG--CCACCCAGGTG---------TCCCATGTT-------------------------
13616 (gray short-tailed opossum) e-value: 1e-46   NNNNNNNNNNNNNNNNNNNNNNNNNNNNNNNNNNNNNNNNNNNNNNNNNNNNNNNNNN
9606 (human) e-value: 3e-111                       AGATGGAGACAGGTGTGTAACTTGTCCAAGGTTGCACAGCGAGGAGGTGCAGAGCTGG
9258 (platypus) e-value: 8e-40                     AATAGGACCCCAG-----AATTGAGGCTATGCT--AGCTA-AGCATTCTCATAGTATA

10090 (house mouse)                                 ----------------------------------------------------------
9615 (dog) e-value: 5e-75                           -----TCTTCCTTTTAATTGGATGTAATTGATATTATACTAGTTAACATCATATTGGT
13616 (gray short-tailed opossum) e-value: 1e-46   NNNNNNNNNNNNNNNNNNNNNNNNNNNNNNNNNNNNNNNNNNNNNNNNNNNNNNNNNN
9606 (human) e-value: 3e-111                       GACTCAAATCC-TAGCAGCCAAACTACCACCCATTATGCCAGAGTACCCGCTCCATTC
9258 (platypus) e-value: 8e-40                     TAGAAAAATCC-----------------------------------------------

10090 (house mouse)                                 ------------------------------------GTTATCACCATGCTCTAGATGA
9615 (dog) e-value: 5e-75                           TAACAGTATAGTAGTCGACATATTCTATAATAATACAAGAT-A---------------
13616 (gray short-tailed opossum) e-value: 1e-46   NNNNNNNNNNNNNNNNNNNNNNNNNNNNNNNNNNNNNNNNNNNNNNNNNNNNNNNNNN
9606 (human) e-value: 3e-111                       CTACTTCCTCTCTACCTCCCCAGTCCCTTCTATTATAGTATAAAACGACTAGAGCTTG
9258 (platypus) e-value: 8e-40                     ------ACTCCATGTCTGCTACTGCTCTT-TTTTAAAAAAAT---C-TGGCATTATGT

10090 (house mouse)                                 GGAAACAG--------------------------------------------------
9615 (dog) e-value: 5e-75                           GTAACGACATTATAGTAGTTGCA-GGTATACAACAGCATG--ATTTGA-TATTTGTAC
13616 (gray short-tailed opossum) e-value: 1e-46   NNNNNNNNNNNNNNNNNNNNNNNNNNNNNNNNNNNNNNNNNNNNNNNNNNNNNNNNNN
9606 (human) e-value: 3e-111                       AAAAAGAGATTTCTGGACAGGCACGGTGGCTCACGCCTGTAAATCCCAGCACTTTGGC
9258 (platypus) e-value: 8e-40                     ACAAATAAATAAATGCA-ATGG--GCCAG-CGAAAGCTATCAATC-------------

10090 (house mouse)                                 ------------------------ATT-------------------------------
9615 (dog) e-value: 5e-75                           GTATTGTGA---AACGACCACGGTAAGCCTAGTTAA---------------CATCTG-
13616 (gray short-tailed opossum) e-value: 1e-46   NNNNNNNNNNNNNNNNNNNNNNNNNNNNNNNNNNNNNNNNNNNNNNNNNNNNNNNNNN
9606 (human) e-value: 3e-111                       ATTTTGGGAGGCCAAGACAAGAGGATTGCTTGAGCCCAAGAGTTCGAGAC-CAGCTGG
9258 (platypus) e-value: 8e-40                     --------------------------------TCTCCCACCCCACAACACACTCCTAA

10090 (house mouse)                                 ------------------------------------------------------GTAC
9615 (dog) e-value: 5e-75                           ---TCATCATG--TA-TAAT-T-ACAGATTTTTTTCTTGCTTTTATTTTATTTATTGA
13616 (gray short-tailed opossum) e-value: 1e-46   NNNNNNNNNNNNNNNNNNNNNNNNNNNNNNNNNNNNNNNNNNNNNNNNNNNNNNNNNN
9606 (human) e-value: 3e-111                       GCGACATAGTGAAAACTTGTGTCTACAAAATAATAATAATAATAAAAATGCCGGGCAC
9258 (platypus) e-value: 8e-40                     ACATGACATATAAAATCAAGGGAGGGAGATATTTAAAGAAATGGGGCCCCTCTAGGTC

10090 (house mouse)                                 AG-GTAACTAAC---TCATCC-----------AAGGTC-ACACAGCTGG---------
9615 (dog) e-value: 5e-75                           TCTATTTATTAATTTTTAGTTAAGTAAGCT-GTACGCCCAAC---GTGGCGCTTGAAC
13616 (gray short-tailed opossum) e-value: 1e-46   NNNNNNNNNNNNNNNNNNNNNNNNNNNNNNNNNNNNNNNNNNNNNNNNNNNNNNNNNN
9606 (human) e-value: 3e-111                       GGTGGCTCACACCTGTAATCCCAGCTCTCTGGGAGGCCGAGGCAGGTGGATCACTTGA
9258 (platypus) e-value: 8e-40                     ACTTCAGCCCAGATATGCCCTGCTTGAGTCTGAAAGAAATAACTGAATGTGGAAGTTT
```

10090 (house mouse) ---------------G---A---------AGCACGG---AGCCAGGACTCTGCTCCTCA
9615 (dog) e-value: 5e-75 GGACCCCGAGATCAAGAGTCGCATGGTCTGCTGACTGAACCAACCAGGCGTCT-----
13616 (gray short-tailed opossum) e-value: 1e-46 NNNNNNNNNNNNNNNNNNNNNNNNNNNNNNNNNNNNNNNNNNNNNNNNNNNNNNNNNN
9606 (human) e-value: 3e-111 GG-TCAGGAGTTCGAGACCAGGCTGGCCAACATGGTGAAACCTTGTCTTTACTGAAAA
9258 (platypus) e-value: 8e-40 GCACCTCAACCT-GAGACCTTTCTTGAAAACA----GAGCTAA-------------AA

10090 (house mouse) T-----------------------G-GCTAAATTGCCTTCC-ACCCT-TCT-------
9615 (dog) e-value: 5e-75 --CACAGATT--TTTTTTG-TGTGATGATAACTTTTAAGATCTACTTATCAATTTTCA
13616 (gray short-tailed opossum) e-value: 1e-46 NNNNNNNNNNNNNNNNNNNNNNNNNNNNNNNNNNNNNNNNNNNNNNNNNNNNNNNNNN
9606 (human) e-value: 3e-111 TACAAAAATTAGCTGGGCGTAGTGGCGCACACCTGTCATCCCAGCTACTCAGGAGGCA
9258 (platypus) e-value: 8e-40 TTTTAAAAGAAGCAGTGTGGCCTAATGAATAGAGCATGAGTCTGGAAGTCA-G-AGCA

10090 (house mouse) -----------------C-----CCCTCAGGCTTTCAAATGCTACCTC-CCTGCTTCC
9615 (dog) e-value: 5e-75 AATGCACAACACAGTAGCATTAACTCT----AGTCACCATGCTGT-AC-ATTACATCC
13616 (gray short-tailed opossum) e-value: 1e-46 NNNNNNNNNNNNNNNNNNNNNNNNNNNNNNNNNNNNNNNNNNNNNNNNNNNNNNNNNN
9606 (human) e-value: 3e-111 GAGGCAC-A-AGAATCGCTTGAACCCAGGAGGCGGAGGTTGCAGTGAG-CCAAGATCC
9258 (platypus) e-value: 8e-40 CCTG--GGTTCTAATC---TC--AACTCCGCCACTAGTCTGCTGTGTGACTTTGGGAA

10090 (house mouse) T-----TCTATTCAAGCGT-----AG-AAACCAG-A---G---C-TTTAAAAAGTGAC
9615 (dog) e-value: 5e-75 ------------------------------CCAGGACTTATTTATTTTATAACAAGAG
13616 (gray short-tailed opossum) e-value: 1e-46 NNNNNNNNNNNNNNNNNNNNNNNNNNNNNNNNNNNNNNNNNNNNNNNNNNNNNNNNNN
9606 (human) e-value: 3e-111 TGCCACTGCATTCCAGCCTGGGTAACAGAGCGAGAACTTGTCTCAAAAAAAAAAAAAA
9258 (platypus) e-value: 8e-40 AGTCACTTAACTTCTCTAGGCCTCACTTATC------TCATCTGTAAAATAATAATTA

10090 (house mouse) TTCTACCAAGAACGTTCAGCAGAATGAG-CCC-AACATAGAACGACAACTCACAGCGA
9615 (dog) e-value: 5e-75 TTTGTGCCTTTGACCACTTTCGCCCGTTTTGCCCACCCTCTATCCCTGC-CTCTGGCG
13616 (gray short-tailed opossum) e-value: 1e-46 NNNNNNNNNNNNNNNNNNNNNNNNNNNNNNNNNNNNNNNNNNNNNNNNNNNNNNNNNN
9606 (human) e-value: 3e-111 TTCTACCAAGAAAATTCATCAGAATGGGCCCCAAACTCAGAATAGTAACTAACA--AT
9258 (platypus) e-value: 8e-40 TTATGGTATTTGTTACATGCTTACTAGGTGCCAGGCACCATAT---TAAGCACTGGAG

10090 (house mouse) CACAAAAGGCCCAAACCAGAGGAAATTACCCTGC------ACAATTTGCAAAT-CACC
9615 (dog) e-value: 5e-75 -ACCACCGATCTGTTCTTGTATCTATGAACTTGGGTTTGTTTGTTCTTAGATTCCACA
13616 (gray short-tailed opossum) e-value: 1e-46 NNNNNNNNNNNNNNNNNNNNNNNNNNNNNNNNNNNNNNNNNNNNNNNNNNNNNNNNNN
9606 (human) e-value: 3e-111 TAAAAAAGACCTAAATCAGGGGAAATGACCATGCTTGAGTACAATTTGCAAAT-CACC
9258 (platypus) e-value: 8e-40 CACCA--TA--CTAAGCACTGGA--T-A-------------------GGGATTTAGAC

10090 (house mouse) -T-------------CAAC-A-----GAGTCTTCTGGGCAAAAT-GAAGGCTAATTTT
9615 (dog) e-value: 5e-75 TGTAAGTGAGATCGGACAGTATTTGTCTTTCTCTTTCTGACTTACTGTACTTAGAATG
13616 (gray short-tailed opossum) e-value: 1e-46 NNNNNNNNNNNNNNNNNNNNNNNNNNNNNNNNNNNNNNNNNNNNNNNNNNNNNNNNNN
9606 (human) e-value: 3e-111 TTGAAA---G-----AGAG-A-----GTATGTCTTCCAGAAAATCGAGAGCCAATTTT
9258 (platypus) e-value: 8e-40 TATGAGTCCCATGTGGGA--------C-ATGGACTGTGTCCAATC-------------

10090 (house mouse) AAAACATCCACATCCTCTGGGCTCCGCCCTTCCCCCCCCACCCAGAGGTTCTTGGGGC
9615 (dog) e-value: 5e-75 ATGCCTTCAAGATCC------ATCCGTGTTGTCACAAATGGCAAGATTTCCTCCTTTT
13616 (gray short-tailed opossum) e-value: 1e-46 NNNNNNNNNNNNNNNNNNNNNNNNNNNNNNNNNNNNNNNNNNNNNNNNNNNNNNNNNN
9606 (human) e-value: 3e-111 AAAACACCCACATCCTCT-GG----G-CCTCTCCCTCCTCCCCAAAGTTTCTTCTGGC
9258 (platypus) e-value: 8e-40 ---TGATTAGCT--T-GT

10090 (house mouse) CAT--TCAAGCAAAAACCCCTCTGCTC--TCATTTGCATTGTTCTTGGGAGGGGGAAA
9615 (dog) e-value: 5e-75 TTATAGCCAAATAATATCCCATTATAT--ATATATATAATAATATT---AATA--TAT
13616 (gray short-tailed opossum) e-value: 1e-46 NNNNNNNNNNNNNNNNNNNNNNNNNNNNNNNNNNNNNNNNNNNNNNNNNNNNNNNNNN
9606 (human) e-value: 3e-111 CTTGCTCAGGCAAAAATCCCTTTGCCT--TCATTTGCATTGTTCTTGGGAGGGGGAGA
9258 (platypus) e-value: 8e-40 -----------ATCTACCCCATTGCCTAGTACAGTGTCTGGTACTTAGGAAGTGCTTA

10090 (house mouse) GTGAGCAAGCTCTGAGGTGTCACTCCCCTGAGAGGTGCAGCAGGGTCACA-C-C--TC
9615 (dog) e-value: 5e-75 ATAAATAACAACTCA-G-G----GCGCCTGGGTGGCACAGTTG-GTT-AAGCAC-CTT
13616 (gray short-tailed opossum) e-value: 1e-46 NNNNNNNNNNNNNNNNNNNNNNNNNNNNNNNNNNNNNNNNNNNNNNNNNNNNNNNNNN
9606 (human) e-value: 3e-111 GTGAGCCAGCTCTGA--AGTCACTCCTCTGAGAAGAGAGCCAGAATCACACCACAGTC
9258 (platypus) e-value: 8e-40 GCAAAT-ACCATTAAAGAAACAAAAAAC--AAAAAAG------AATCACA--TC----

10090 (house mouse) GTTTGGCACAACCGGATC-AGGCGTGAACCTGTGCAAAGTTGGCCTACAG-TCCAAAT
9615 (dog) e-value: 5e-75 ACCTT---GATTTTGACTCAGGTTATGATCTCAG-GGTCATGAGATTGAGCCCCACTT

```
13616 (gray short-tailed opossum) e-value: 1e-46    NNNNNNNNNNNNNNNNNNNNNNNNNNNNNNNNNNNNNNNNNNNNNNNNNNNNNNNNNNNN
9606 (human) e-value: 3e-111                         AGTGTGACCAGCGTGACCTCCGTGGGAATTTGGCTGAAGCATCCAAAATGAGTCAAAT
9258 (platypus) e-value: 8e-40                       -----------TTTGAATTGCCTTCTCCAGCTCCTATTGGTCTGATTTGCATTTACTG

10090 (house mouse)                                  GAGTTAATTAGTCAGCATTGCCCCCAGAGAAGGTAGACGATTGACCTTTCCCAAGAAA
9615 (dog) e-value: 5e-75                            G-GG---GCTCTGGACTCAGCAGGGAGTCTACTTGAGATCTTCTCTTTCCTCTGACC
13616 (gray short-tailed opossum) e-value: 1e-46    NNNNNNNNNNNNNNNNNNNNNNNNNNNNNNNNNNNNNNNNNNNNNNNNNNNNNNNNNNNN
9606 (human) e-value: 3e-111                         G----AATTAATCAACTCTTCCCCTAGAGAATGCGGACACTTGATCCTTCACAAGAAA
9258 (platypus) e-value: 8e-40                       CAGAAAATTGTTGGTTCTAGGGAAGA--------------AGAACTATTCCT------

10090 (house mouse)                                  ATGAACGTGCCCCACCCAGTGACAAGAAATGAGGGCACCGG-T----CATGCGATAAA
9615 (dog) e-value: 5e-75                            CTCCGGTGCTTGCTTGCTCTCTC-TCTAA--A----AAA--AAAACAATAATAATTTA
13616 (gray short-tailed opossum) e-value: 1e-46    NNNNNNNNNNNNNNNNNNNNNNNNNNNNNNNNNNNNNNNNNNNNNNNNNNNNNNNNNNNN
9606 (human) e-value: 3e-111                         ATGAGGATGCCCCACCCAGTGGCAAGAAATGAGGGCACTGGTTACCAAAAGGAAAATA
9258 (platypus) e-value: 8e-40                       -----------------------CTGTATTT-----------------TCTGGATA--

10090 (house mouse)                                  AACACAGGCCTGAGAGAGCTATAGTAAGAGCT----CC-TGCTGTGTTGGACCCT--G
9615 (dog) e-value: 5e-75                            AAAAAAATAAGAAAACATCTCTATCCATCCATCCATCCATCCCTCGATGGACACTTGG
13616 (gray short-tailed opossum) e-value: 1e-46    NNNNNNNNNNNNNNNNNNNNNNNNNNNNNNNNNNNNNNNNNNNNNNNNNNNNNNNNNNNN
9606 (human) e-value: 3e-111                         GAAAAATGGGAAGAACAGTGTTTTAAAGTGCA----TC-TACCTTGTTTGACCCTGAG
9258 (platypus) e-value: 8e-40                       ----------AAATACAGAGTTAGAAA-----------------------AGTCTCTT

10090 (house mouse)                                  --TGC-T-----------GTGGGGTGTGCTGGGCACCATCA-TGATCAAGGTGCATGA
9615 (dog) e-value: 5e-75                            GCTACTTCCATATCTTGGCTATTGTGAACAATGCTGCAACGAACATAGGGGTATATTT
13616 (gray short-tailed opossum) e-value: 1e-46    NNNNNNNNNNNNNNNNNNNNNNNNNNNNNNNNNNNNNNNNNNNNNNNNNNNNNNNNNNNN
9606 (human) e-value: 3e-111                         CTTGGGTGGCAGACCCAGGTGGGTCTGCCAGGCCACCATCATTTGTCAAACTACATTC
9258 (platypus) e-value: 8e-40                       TTACTTCAGATAATTCAAGGGAAAACAAATTCTTACTGACCAGTTCTGAGTCCCAAAA

10090 (house mouse)                                  CCCCTGGAGATTTGGGGAGGCCCTGTATGGTGAAGTGTGGCAGCTGACAGC-ATTTGG
9615 (dog) e-value: 5e-75                            ATTTTCAAGTTATTGTTTCTATTTCTTTTGGGTAAATACGAGTGGAATTGCTATCTTT
13616 (gray short-tailed opossum) e-value: 1e-46    NNNNNNNNNNNNNNNNNNNNNNNNNNNNNNNNNNNNNNNNNNNNNNNNNNNNNNNNNNNN
9606 (human) e-value: 3e-111                         ACCCTGGACATTCAGGAAGGCCCTAAAACTGAAACAGCATCTCCCAACAGC-ATCTGG
9258 (platypus) e-value: 8e-40                       ATAAAGGAGAGAAGGAAAAGAAAAATGTAAAATATATCTCCCACATACTCTGCAATTG

10090 (house mouse)                                  -GAAACATGGAGAA--------------------TTTCCCCA--G---GTTTATACAG
9615 (dog) e-value: 5e-75                            AGCATTTTGAAAAACCCCCAT--------ACTGTTTTCCATAGTG-------------
13616 (gray short-tailed opossum) e-value: 1e-46    NNNNNNNNNNNNNNNNNNNNNNNNNNNNNNNNNNNNNNNNNNNNNNNNNNNNNNNNNNNN
9606 (human) e-value: 3e-111                         AGGACCACAAAGAATTTCTGTGGGGCTACATCATTTTCCCCAGTGTTTCTCTATGCCT
9258 (platypus) e-value: 8e-40                       GGTTAAGCAAACATGGGACCAAGTTTACTCTGCATGCCCCA--------TCTTATAA

10090 (house mouse)                                  CAGTTCACTCATCTATTTTAAAATCC-----------------TGTTTATTTATTTAA
9615 (dog) e-value: 5e-75                            ------GCTGCACCAATTTA-CATCC--------------------------------
13616 (gray short-tailed opossum) e-value: 1e-46    NNNNNNNNNNNNNNNNNNNNNNNNNNNNNNNNNNNNNNNNNNNNNNNNNNNNNNNNNNNN
9606 (human) e-value: 3e-111                         TGCAAAGCAGTTGATTATGAGGATCCTAGGCTCTTTTTTTTTTTTTTTTTTTTTTTAA
9258 (platypus) e-value: 8e-40                       CACCAAACAAATGTTTTGTATAATGGATTGACTATTATTTGTGAGCTATTCAATATCT

10090 (house mouse)                                  TGTGTGTGTGTGTGTGT-GAACATCTGTGCTGCTGTGTATGTATGTGTGTGGAGATCA
9615 (dog) e-value: 5e-75                            ----------------T--------GATGTTTTAACACCCCATTGGACTTGGAGCTCC
13616 (gray short-tailed opossum) e-value: 1e-46    NNNNNNNNNNNNNNNNNNNNNNNNNNNNNNNNNNNNNNNNNNNNNNNNNNNNNNNNNNNN
9606 (human) e-value: 3e-111                         GGGATGGGGGTCTCACTCTGTCTCCAAGGCTGGAGTGCAGTAATGCAATCATAGCTCA
9258 (platypus) e-value: 8e-40                       TGTAT-----------------------------------------------------

10090 (house mouse)                                  ---GAGAA-CAACTTTTGGGAGTTAGTTCTCTCCTTCC-------ACCAT--ATGTGC
9615 (dog) e-value: 5e-75                            GGGCACCATAGTCAGTCCAGGTCCCTGGGCCACCTTCCCCCGTCAGACCACTTTTACC
13616 (gray short-tailed opossum) e-value: 1e-46    NNNNNNNNNNNNNNNNNNNNNNNNNNNNNNNNNNNNNNNNNNNNNNNNNNNNNNNGGCC
9606 (human) e-value: 3e-111                         CTGCAGCCTCAAAATCCTGGGCTCAG--GGGACCTTCCTGCCTCAGTCTTCCAAGTAG
9258 (platypus) e-value: 8e-40                       -------CCCAATAGTCCCAAAAGAGTCTGTGACTCTCTCACCCCATATTCTAACCCT

10090 (house mouse)                                  CCC-----GAG-----------------------------------------------
9615 (dog) e-value: 5e-75                            CTGAGATTCAGG-------GA-CGT-CCTCAC--CCT-------------GGGAAATG
13616 (gray short-tailed opossum) e-value: 1e-46    CTGGAGGCCATTGAT-TT------------AATCTC---AGCTTTGGTGGAGATTAAA
9606 (human) e-value: 3e-111                         CTGGACTACAGGTATGTACCACCATGCCTAGCTATTTAAAAAAATTTTGGAGAGATG
9258 (platypus) e-value: 8e-40                       CTGTCTTTGA--------CTGGCACTGAAGAATCACAAATGGATTATTTGAAGTGAAG
```

```
10090 (house mouse)                                  ------------------------------------------------GAT-------
9615 (dog) e-value: 5e-75                            TTGCCT------------C---CCGACTGTAGCC-GAGAGCCACCGGGAATCC-----
13616 (gray short-tailed opossum) e-value: 1e-46     GCCACCTCTTTCAGTGACCCACCTCCCTGAGTCTCCCCACTTTCCCTGGATCC-----
9606 (human) e-value: 3e-111                         GGGTCTCACTATATTGCCCAGGCTGATCTTGAACTCCTGGCCTTAAGGTATCCTCCCA
9258 (platypus) e-value: 8e-40                       GTGT-TT--TCTATT------G------------------------TGTTTCC-CCTG
```

```
10090 (house mouse)                                  ---TAGACTCTTCATCAGGCTTGGCAGCAAGTGCATTTACCCACTGAGCCATCTCACT
9615 (dog) e-value: 5e-75                            ------GTGTGGGGCGAGGTCATTCTCCAAAGGCCTTCTCTGTGCCCTGCAACGCGGT
13616 (gray short-tailed opossum) e-value: 1e-46     -----------------------CCCACCAGGGCCAATGGTGTGGTGAGAGGCCCGTT
9606 (human) e-value: 3e-111                         CCTCAGCCTCCCAAAATGCTGGGATTGCAGGTG-----------TGAGCCACCGCCCT
9258 (platypus) e-value: 8e-40                       AGTCCTCCTGGCAGTGAGAGTTAATTATAAGAACC-T----GAAGGCTATAATATGTT
```

```
10090 (house mouse)                                  GACCGAAGAGCAGTTTATTATAGTGATCAT-GAATGTCCCACATATGACCCTGAGGTC
9615 (dog) e-value: 5e-75                            TTATTATAAGGATCTGAGGTTCCT-AAAATCCTAGCGCCCTGGGATGATCTAGAGGTC
13616 (gray short-tailed opossum) e-value: 1e-46     TGATAGGAGGAAGCAGA-----------------------------------------
9606 (human) e-value: 3e-111                         --CTGCCAAGGATCTGAGGTTCGTGAATATCCCAGAGCCCTGATACAATCTAGAGGTC
9258 (platypus) e-value: 8e-40                       GGAGCCCAGGGAGCT----CTAGTGACTGCTGCTTTGTCCCGTTAATGTTTGATGGCA
```

```
10090 (house mouse)                                  AC--TGTACCAGCATTTCCCAAC-GC--CTTCCCTTGAGTCA---C-TT---TGGGA-
9615 (dog) e-value: 5e-75                            ACGCTGTATTAACTTTTTCTAATTCCTTCTTCTCTTGAGCCGCCTCACAGAGTGCTTT
13616 (gray short-tailed opossum) e-value: 1e-46     ----------------------------------------------------------
9606 (human) e-value: 3e-111                         ACATTGTATTAACTTTTTCTAACTCCTTCTTCCTTTGAGCCACCTCATGAAGTGCCTA
9258 (platypus) e-value: 8e-40                       ACATTTTA-----------GAGAGGCCTTTTCCTCTGACCCTAACAGAAGGAGGCATT
```

```
10090 (house mouse)                                  CTTGTCTGTGGGTCTCT-GTCT----------------TAGGTCAATCC--C-CAAAC
9615 (dog) e-value: 5e-75                            GTCGTCTGTGGGTCCTTGTTCCAGGCTGCA-GGGGGTCCTGCTCAATCCCAAATCAT-
13616 (gray short-tailed opossum) e-value: 1e-46     ------------------------------------CTGTGACTCATCC-------AG
9606 (human) e-value: 3e-111                         CTTACTTGGGGTTTCCA-TTCTAGGCTGCA--GGGGTCCTACCCATTCCCACATCATC
9258 (platypus) e-value: 8e-40                       GCTTGTTCAGAGACCCTGGTCAATAGTGCACTGGAGTCTTGATTAGGATTATAAAATG
```

```
10090 (house mouse)                                  A---TTCCTTGTCCCAGAGTCCTGGGGATGGTC-A---GATAATCATGACTTTTTTTT
9615 (dog) e-value: 5e-75                            -CCGTTCC--CCACCAGGGGCATG-GGGTGGCCTG---AATAGCCATGAC---TTTTG
13616 (gray short-tailed opossum) e-value: 1e-46     GGCTTTCC------------T-GGGGGATTCTCTGTGTGATA--------------AC
9606 (human) e-value: 3e-111                         ACCTTTCC---ACCAAGGAATCTGAGGGTGACCTG---GATAGTCATGA-----TTTT
9258 (platypus) e-value: 8e-40                       ACAATTTGGAGAGACTCCTGTGGGAGGCTTTGTAT---GATAGCTCAGA---------
```

```
10090 (house mouse)                                  TTTTAATCAGGAGAATTAAAATAATTTTTACCTCCAGGAAATTTCCAAACCAGGC--A
9615 (dog) e-value: 5e-75                            T-TT-AGCAGGAGAATGAAAGTCATTTTTATCTCCGCGAAATGTCAAG-CCGGGC--A
13616 (gray short-tailed opossum) e-value: 1e-46     CCTTAAGCAAGAAAATGAAAACAATTTTCATCTCTGTGAAATTTCAGCCCCAAACAGA
9606 (human) e-value: 3e-111                         TATTTAGCAAGACAGTGAAAATAATTTTCATCTCAATGAAATTTTAAGACCAAGC--A
9258 (platypus) e-value: 8e-40                       ------GTGGGATAAAGAAAACAATTTTCACCTTCAGGAA-TTTCAAAACAAAGC--A
```

```
10090 (house mouse)                                  AAA--------------TGTTCTTGGCTTTCCACGGCCTCCTGGG----------GGC
9615 (dog) e-value: 5e-75                            GAA--------------TGTGTGTGGCTCTGGCTGGCCTCCTGGGCCCCCCCACCCCC
13616 (gray short-tailed opossum) e-value: 1e-46     AAATGGTGGCTCTCTTTAGGTTATTTGAGCCCTTGTGATTTTGGAGCAGGCAGAGAAG
9606 (human) e-value: 3e-111                         GAT--------------TGTGCATAGCCCTCCTTGGCCTCCTGGG----------ACC
9258 (platypus) e-value: 8e-40                       AAA-----------------------CAAATGTGCAGGTCCAGGGGGAGAAAAACA-C
```

```
10090 (house mouse)                                  CCCGGGCTCTGT-G-TCC-C-TGAGCTGTAGGGAGGATAGCATGCCT-GTGTGCAGTG
9615 (dog) e-value: 5e-75                            CCAGGCCTCCGT-GCCCCCCC-----ACCCCCCAGCGTGACTCTGCA-GCGT---G-A
13616 (gray short-tailed opossum) e-value: 1e-46     AGGGCCCAGGTGGGGGGGAGGGGGGAAGGCCAGAGGATGG--GGGGGGGTTTGGAAGT
9606 (human) e-value: 3e-111                         CCAGGCCACTGTGGCCCCACTAGCAGGGAGGAGAGGATGACTTTGCA-ATGT----AA
9258 (platypus) e-value: 8e-40                       ACAAAACTTTATAATCTTCTAACTGGCAAGAGGGGGAAAACAGGGTACAGGTGAAGGA
```

```
10090 (house mouse)                                  GGGCTATTCGTCATCA---G-------------AAC-A---ACAAGGCCAGCAGCAGT
9615 (dog) e-value: 5e-75                            GGGCCATCCGTCACTG---G---------AACAAACAAGGCACGAGGCCCTCGGCAGC
13616 (gray short-tailed opossum) e-value: 1e-46     TGGGGGGCAGGGATGAAAGGGGTGGCTTCTCCCACAATAGTAGAGTCCCAGCAGCCCC
9606 (human) e-value: 3e-111                         GGGCTATTCGTCATTA---G-------------AAC-A---ACAAGGCCAGCAGCAGC
9258 (platypus) e-value: 8e-40                       TGTACTTTCCTCATGAGCTGCATATTAGTCACTAAGATAATACAATGAC-TAGTTGCT
```

```
10090 (house mouse)                                  GTGGCTCTCACCTTATCAGGGTGGTTGGCCTAGCTGGATTCTATTAATTGCTGAAGGC
9615 (dog) e-value: 5e-75                            GCTGCTCCGGCCTTATCGGAGCGGGAGGCCTAGCTGGATTCTATTAATTGCCCAATGC
13616 (gray short-tailed opossum) e-value: 1e-46     GTGGCT------TTATCAGAGTGGGAGGAATAGCTGAATTCTATTAATTGCTGGGTCC
9606 (human) e-value: 3e-111                         ATGGCTCTGGCCTTATCGGAGCAGGAGGCCTCGCTGGATTCTATTAATTGCCGAATGC
9258 (platypus) e-value: 8e-40                       TTTTCTGTTGTTTTATCATAGCAGGATGAATAGTTAAATTCTATTAATTGCCGAATGC
```

10090 (house mouse)	-TGTTGGAGTTGGCAGATACAGGAAACAGTTTACATGGTGACACAGGGCCCCAGCTCG
9615 (dog) e-value: 5e-75	-TGTTGGGTTTGGCAGATACAGGAAACAGTTTACATGGCTACACGGTGCCCCGGCTCA
13616 (gray short-tailed opossum) e-value: 1e-46	ATGTTTGACTTGGCAAATACAGGAAGCAGTTAACATGGCTACAGAGGGCTCTGGCTCC
9606 (human) e-value: 3e-111	-TGTTGGGTTTGGCAGATACAGGAAGCAGTTTACATGGCTACACAGTGCCCTGGCTCA
9258 (platypus) e-value: 8e-40	-TATTTTGTTTGACAAATACAGGAAGCAGTTTACATGACTACGCAGTGCTCTGGTTCA

10090 (house mouse)	CAGCCTCTCAGAAACTGTATTATTGTAGTGGCATAATTGTGATTAGGGACAGTTCCCT
9615 (dog) e-value: 5e-75	CAGTCTCTCTGAAATGGTATTATTGTAGCGGGATAATTGTGATTAGGGACAGTTCCCC
13616 (gray short-tailed opossum) e-value: 1e-46	CAGTCTCTCTGAAACTGTATTATTGTAAGGGGATAATAGTGATTAGGGACAGTTCCCT
9606 (human) e-value: 3e-111	CAGTGTCTCTGAAATGGTATTATTGTAGTGGGATAATTGTGATTAGGGACAGTTCCCC
9258 (platypus) e-value: 8e-40	CAGTCTCTCTGAAACAGTATTATTGTAACGGGATAATAGTGATTAGGGCCAGTTCCCT

10090 (house mouse)	GAGGAAGAGCAGACCTTTGATGATTGAGTCACTTCC--TCTGCACTCCACTTCA-GTG
9615 (dog) e-value: 5e-75	GAGGAAGACCAGGCCTTTGATGATTGAGTCACTTCC--TCCCCACTCGGCTTCA-CTG
13616 (gray short-tailed opossum) e-value: 1e-46	GAGGAAGGAAAGCCCTTTGATGCCTGACTCACTTCCTTTCTCTGTTCTACTTCAGGGG
9606 (human) e-value: 3e-111	GAGGAAGACCAGGCCTTTGATGATTGAGTCACTTCC--TCTCTACTCTACTTCA-GTG
9258 (platypus) e-value: 8e-40	GAGGAAGGACAGCCCTTTGATGATTTAGTCATTTCCTTTCTGTGCTCTACTTCA-ATA

10090 (house mouse)	-TGATAGTGT-G---G-----C---CCCACGGACCCTTGGCTGGCCAGAG-ACTTTAG
9615 (dog) e-value: 5e-75	-TGATAGCGCAGCTCGGCCCACAGACACAC--ACCCTTTGCTGGCCAGGGCGGTCCCC
13616 (gray short-tailed opossum) e-value: 1e-46	CAGATAGAGCTTCACTACACACACACACACACAC----------------ACACAC
9606 (human) e-value: 3e-111	-TGATAGCGTGGCTCGCCCCACAGACACACACACCCTTTGCTGGCCAGAG-AGCCCTG
9258 (platypus) e-value: 8e-40	-AGATAGAGCTTCTCTCACCAGAGACACAC--ACCCTTTTGTAGCCATAG------AG

10090 (house mouse)	AGTCCCACAGGAAAGCTCTCCAACAGGGGCAGTCCAACCTC
9615 (dog) e-value: 5e-75	GGGTCCGGGGCCAGGCTCCCCGGGTGGGCAGGAGGGCCTG-
13616 (gray short-tailed opossum) e-value: 1e-46	ACACACACACACACACTGAAAAACTGAAAGTTAAGACCAT-
9606 (human) e-value: 3e-111	AGTCCCACAGCAGGGTTCCCAAACCAAGAGCCAAGAGCTGC
9258 (platypus) e-value: 8e-40	TCCTCCAGGAGAGAGTTCAAAAGCTTATGCCCTCCA-----

D

Lmo2 -75kb: Manual alignment

```
mouse    : CTTCTTGGGGCTTTCTTGGATCCCTCCAGAACTTTCCTCTTTTTGATTTTTTT--TATAAAGGCAAAAATTTAATTGGGAATAACTTACA
human    : ------------------------------------------------------------------------------------------
dog      : ---------------------------GATATTAGCTGGAGAGTGAAGGACAG--CACAAAACACCGAAGTAACCCGAGGCTCAAGTTT-
opossum  : ----------------------------------------------------------------------GTGTGTGTAGTGAAGCTCTA
platypus : --------------------------TTGAACTCTCTCCTGGAGGACTCTATGGCTACAAAAGGGTGTGTGTCTCTGGTGAGAGAAGCTC

mouse    : GATGCAATAAGCATCCATTATTATTATGGCATCATGCAGGCAGACTTGATGTTTGAAGAGCCTCCAGCCTTGATTCAAAGACAGCCAAGA
human    : ------------------------------------------------------------------------------------------
dog      : -GTGTGATAGTCACTATTGTTCAGTATGTCGCTGGGCCTTGGGATTTGGATT----AAAATCTTCATCCGGTAGCAAGGGAAAGTTGAGA
opossum  : TCTGCCCCTGAAGTAGAACAGAGAAAGGAAGTGAGTCAGGCATCAAAGGGCTTTCCTTCCTCAGGGAACTGTCCCTAATCACTATTATCC
platypus : TATCTTATTGAAGTAGAGCACAGAAAGGAAATGACTAAATCATCAAAGGGCTGTCCTTCCTCAGGGAACTGGCCCTAATCACTATTATCC

mouse    : GGA-TGATATCTTCTGCATTGGGTAGAGCTTGAGTATAGGACCTCAAAGCCCATTCCCACAGTGACTCACTTCTTTCAACAGGCCACACC
human    : ------------------------------------------------------------------------------------------
dog      : TTAGCCATGGCCTTTGAACTAGATGTGACAAAGTTGAGACTGAAGATTATCTGCAGAAGCAGCTAACTGTTGTGTGTAGAGGAGCGAGGG
opossum  : CCTTACAATAATACAGTTTCAGAGAGACTGGGAGCCAGAGCCCTCTGTAGCCATGTTAACTGCTTCCTGTATTTGCCAAG---TCAAACA
platypus : CGTTACAATAATACTGTTTCAGAGAGACTGTGAACCAGAGCACTGCGTAGTCATGTAAACTGCTTCCTGTATTTGTCAAA----CAAAAT

mouse    : TACTCTAACAAGGCCATATATACCTCCTAATAGTGCCACTTTTCCACTTCCCCTGGGCCAAGTA-TATTCAAGCCAC----CATAGCACC
human    : -----------CTCCTTGGGTGCTTTCT--CAGCAACGCCTTCCCTGCTTCCCACAGTCCTGGA-TGGGATTCTTAG----CAAATCACT
dog      : GAAGGTTCAAAGACTGGAGCATTCCTTGCCCAGATTCCAGATGCTCACACAACAGGGCCCCGGA-GATTAGTGCTGGTCTTCGTGTGGCT
opossum  : TGGACCCAGCAATTAATAGAATTCAGCTATTCCTCCCACTCTGATAAAGCCACGGGGCTGCTGGGACTCTACTATTGTGGGAGAAGCCAC
platypus : AGCATTCGGCAATTAATAGAATTTAACTATTCATCCTGCTATGATAAAACAACAGAAAAAGCAACTAGTCATTGTATTAT-CTTAGTGAC

mouse    : CCTCAAAGAGCTGGTT--GCAGTTTG--ATTTT-TATTAGTA-CTGATTTGCCTTTTGTTTTCA---CTTTGAGTACA---CTCTGAACT
human    : CCTTCAGAACTTTGCT--CCCTTTCT--TTGTAACATTTGAT-TTGGTTTGTTGTTCTGTACCT---ATTTTAGTA-A---TTCTTTAAT
dog      : CCTCAAGGCCATTATCTAGCGGTTAT--CTGTCCCACCTG---CTGCCTGGCCTTGGGGAGGCTGAGCCACAGCTCCA---CTTCGGAGA
opossum  : CCCTTTCATCCCTGCCCCCCAACTTCCAAACCCCCCCCCATCCTCTGGCCTTCCCCCCTCCCCCCCACCTGGGCCCTCTT-CTCTGCCTG
platypus : TAATATGCAGCTCATGAGGAAAGTAC--ATCCTTCACCTGTACCCTGTTTTCCCCCTCTTGCCAGTTAGAAGATTATAAAGTTTTGTGTG

mouse    : TATTGAGTGACACTG----------ACCTCCAGT-------CTTCTAGTG----ATCTTAAACTTGG-TCAAGG-GA--GAAATCTCTCT
human    : TAATGTATGTCTCTA----------CCACTTAGA-------TCTTAAGC---------TCAGCCAGG-GCAGGA-ATCTGCAATCTGCCT
dog      : GGCCCAGTCATCTTT----------CCATTCAGG-------CTACCTCCA----G---TACTTCTGG-TGAAGG-AG--ATGTTCCACCA
opossum  : CTCCAAAATCACAAGGGCTCAAATAACCTAAAGAGAGCCACCATTTTCTGTTTGGGGCTGAAATTTCACAGAGATGAAAATTGTTTTCAT
platypus : TTTTTCTCCCCCTGG----------ACCTGCACA-------TTTGTTTTGCTTTGTTTTGAAATTCC-TGAAGGTGAAAATTGTTTTCTT

mouse    : GGTTTTGCTTG-CTACTATAGTTCCATGCCAACTACAGTGCTTAGCACACAGTAGGGACTCATAAG-TAAGTTACCAA------------
human    : GGTTTTGCTCA-CCGTGATAGCCCCATGCCTACCACAGTGCCTGGCACACAGTAGGAACTCAGACA-CAAGTTGTTGAATGAATATATGG
dog      : GTCACAGGACA-TTGGGGGTGCGCT-TGATATCTGGGCCAGCTCCCATGGAGCAGCCACTTCCAGC-TGTTTCGGTGC-----CATTGAC
opossum  : TTTCTTGCTTAAGGGTTATCACACAGAGAATCCCCCAGGAAAGCCCTGGATGAGTCACAGTCTGCTTCCTCCTATCAAACGGGCCTCTCA
platypus : TATCCCACTCT-GAGCTATCATACAAAGCCTCCCACAGGAGTCTCTCCAAATTGTCATTTTATAAT-C--CTAATCAAGACTCCAGTGCA

mouse    : -----------------TAGATAAGTTCATGGCTGGCTGGCTGAATAAATGAATGAATGGATATTTTATAAGTGGTCAAGG-ATGCAAAA
human    : ATGGATGGATGGATGGATGGATGGATGGATGGATGGATGGATGAATGAATGAACGAACGAATGCTTTCTAAGTGCCCAGGATATAAAAAG
dog      : GTGGAAATTCACCTCAGTAACAAAAGACGTGAA-ACTTAGCAATGGAATTGAGTCTTCCAGTTTCTTTGAACTCACCCTTCCACTAGAGA
opossum  : CCAC--ACCATTGGCCCTGGTGGGGGATCCAGGGAAAGTGGGGAGACTCAGGGAGGTGGGTCACTGAAAGAGGTGGCT-TTAATCTCCAC
platypus : CTATTGACCAGGGTCTCTGAACAAGCA-ATGCCTCCTTCTGTTAGGGTCAGAGGAAAAGGCCTCTCTAAAATGTTGCCATCAAACATTAA

mouse    : C--ATCAAAGTAACAT---GCTTAAAGGTACACCATAGCAAAG-CTGGT--TCTATATGACAACG-AC-CAAGCGG---AGGCTGTATTA
human    : CTAGACAAAGTAACTTAAGGCTCAAATATGCGTGATAGTCATG-ACTGT--TCAATATGACTTCGGAC-CAAGGAATTTGGACTGAAATG
dog      : ---GAAAGAGATGCAT---GCGTGAGGGGGACAGGTTCCAATG-AGAGTAGCCAGTCTGGTGAAGGACTCAAGCTTTCTGGGTGGAGACG
opossum  : CAAAGCTGAGATTAAAT---CAATGGCCTCCAGGGCCNNNNNNNNNNNNNNNNNNNNNNNNNNNNNNNNNNNNNNNNNNNNNNNNNNNNN
platypus : CGGGACAAAGCAGCAGTC-ACTAGAGCTCCCTGGGCTCCAACATATTATAGCCTTCAGGTTCTTATAATTAACTCTCACTGCCAGGAGGA

mouse    : TTACATTAGGTAG-AACTCAAAGC----CAAAGTCAACCATTCCCTTTGAACTCA-GTGTGATAGAGTAGAGTTGAGCCATTATCTTCTG
human    : CT-CATTAGAGAGCAACTAAAAGC----CAAAGTCAGCTATTCCCTTTGAACTAG-GTATGACAAAGTTGGACTGGATATTATCTTCTG
dog      : TC---TCAGAAGGGATGTGCCAGT----TCTGTTCAAATATTTGAAGCATTTCCACATAGACCGCCACAGAGATCGGACTTAAATTTCCT
opossum  : NNNNNNNNNNNNNNNNNNNNNNNN----NNNNNNNNNNNNNNNNNNNNNNNNNNNNNNNNNNNNNNNNNNNNNNNNNNNNNNNNNNNNNN
platypus : CTCAGGGGAAACACAATAGAAAACACCTTCACTTCAAATAATCCATTTGTGATTCTTCAGTGCCAGTCAAAGACAGAGGGTTAGAATATG

mouse    : AGGCAGCTAACTGTTACAGGAGAAGGAGGGCTGAGGAGGGGGAAGGGGGGTGGCTCAGGCCCAAATTAAGTGCCTCAGAGGCTGT--AGT
human    : AAGCAGCTAACTGTTACATGAAGACAAGGG-----------AGTGGGAGGTCTCCTGGGCCCAAATCAAATGCATCAAAGACCAT--AGT
dog      : ACGGTTCTGGAGAGCACAGCAAGAACCAAAC---GATGGCTCCAGAGACACAACCTTGGACTCAATGACAGCAAGAAAGGTCCATCAACC
opossum  : NNNNNNNNNNNNNNNNNNNNNNNNNNNNNNNNNNNNNNNNNNNNNNNNNNNNNNNNNNNNNNNNNNNNNNNNNNNNNNNNN--NNN
platypus : GGGTGA--GAGAGTCACAGACTCTTTTGGGACT-ATTGGGATACAAGATATTGAATAGCTCACAAATA-ATAGTCAATCCATTAT--ACA

mouse    : ATACTCTCCC-AGATCCCAGATGCTCACACAACCTGGCCTGAGAGATTAGAGCTGGTCTTCCTGTGGCTCCCTG--------------CT
human    : GTCCCTTCCCCAGATCCCAGATGTTCACACAACATGGCCAGAGAGATTAGTACTGGTCTTCCTGTGGCTCCCTGATGCTGCTATCCAGCT
dog      : AGAACTTTCTGAAA----AGGAAATGAGCCACCTTTCTGGAAGTGTTCAAGCCTGGCCTGGGTATGGTCGTTTATTCCATTCAG--ATAT
opossum  : NNNNNNNNNNNNNNNNNNNNNNNNNNNNNNNNNNNNNNNNNNNNNNNNNNNNNNNNNNNNNNNNNNNNNNNNNNNNNNNNN-----NNNN
platypus : AAACATTTGTTTGGTGTTATAAGATGGGGCA---TGCAGAGTAAAACTTGGTCCCATGTTTGCTTAACCCAATTGCAGAGT-----ATGT

mouse    : GTTATCTGACCCAACCTGCTGCCTGACATTGCAGGAGGCTGAGTCTGGGCCCCACCTCAAAGGGGCCCTA-CATCTTCTCAGTAAAACTA
human    : GTTATCTGGCCCAACCTGCTGCCAGGCCTTG-GGGAGGCTGAGCCACAGCCCTGCCTCAGAGGGGCCCTGTCATCTTTCCATTAAAGCTA
dog      : GTAATGAGGGATGTTCCCTAGGCCTGCTGTTTGTGAGAAAGAGCTGGATGCTGGGAGTAGGGGAGAGAGAGAATCTAATATGACAAACAA
opossum  : NNNNNNNNNNNNNNNNNNNNNNNNNNNNNNNNNNNNNNNNNNNNNNNNNNNNNNNNNNNNNNNNNNNNNNNNNNNNNNNNNNNNNNNNNN
platypus : GGGAGATATATTTTACATTTTTCTTTTCCTTCTCTCCTTTATTTTTGGGACTCAGAACTGGTCAGTAAGAATTTGTTTTCCCTTGAATTA

mouse    : CTTCCAGCTCATGCGTGGGGTTGGAGTGAAAGTGTC--CCACTGACGATAGAGGAGGAGAG---GATTGGTCTCT-----AGAATGACCT
human    : CCTCCA--TTACTTCTAGTG-----ACGGAAATGTT--CTACCAGCTACAGGGCATTGGGGTGTGCTTAGTCTCC-----AGAACCACCT
```

```
dog      : TTAAAAGCCATTCATTAGTAGCTCAATATGTGGGTTT-CCAGCACTTACATGCACCAAGGACCGAACTAACTACTTTGTGGGCATGACCA
opossum  : NNNNNNNNNNNNNNNNNNNNNNNNNNNNNNNNNNNNNNNNNNNNNNNNNNNNNNNNNNNN-----NNNNNNNNNNNNNNNNNNNNNNNNN
platypus : TCTGAAGTAAAGAGACTTTTCTAACTCTGTATTTTATCCAGAAAATACAGAGGAATAGTT-----CTTCTTCCCTAGAACCAACAATTT

mouse    : CCTGTCTGTCACCTAACCCTCATGGTTTAGGTGCCACAGGAGTGGAG----ACTTA------------------GAAATGTGCAACGTA
human    : CTCATTAATCAGCCACCTCCAACCATTTTGGTGCCATAGGGCAGAG----ATTCACCTCCGTAACAAAATATATGAAACTTGGATGGTG
dog      : CTTAATCCTCATGACAATCTTGGGCATTAAGTCCTACAATTATCACCCT--GTTAGAGATCGGGAGACTGACACTGAGAGGAGTGAAATA
opossum  : NNNNNNNNNNNNNNNNNNNNNNNNNNNNNNNNNNNNNNNNNNNNNNNNNNNNNNNNNNNNNNNNNNNNNNNNNNNNNNNNNNNNNNNNNN
platypus : TCTGCAGTAAATGCAAATCAGACCAAT-AGGAGCTGGAGAAGGCAATTCAAAGATGTGATTCTTTTTTGTTTTTTGTTTCTTTAATGGTA

mouse    : TTCCCTGCTAACTGGGTGAGCCTTCTGATGTCTTAGATCTCAGCGTCCCGT------GAAATTGA---GTGGGATAACTTCCACAGTCT
human    : TTTCCTGTTAAACGAATGAGTCTTCTGATTTCTTTGATCTCACCCTCCTACCAGACAGAAATTGATAAATGGGGACATGTTCCAATGAGA
dog      : ATTTGCCCCAAGTCACACAGCGAGGAAGTAC---AGAGCTGGGACTCAAATC-----CTAACCACCAAGCTATTACCCATTCTGCTA-CA
opossum  : NNNNNNNNNNNNNNNNNNNNNNNNNNNNNNN---NNNNNNNNNNNNNNNNNNNNNNNNNNNNNNNNNNNNNNNNNNNNNNNNNNNNNNNN
platypus : TTTGCTAAGCACTTCCTAAGT-ACCAGACAC---TGTACTAGGCAATGGGGTAGATACAAGCTAATCAGATTGGACACAGTCCATGTCCC

mouse    : ACGGCACACCTCA-GGAAAGCGCTCTCAACTATTCCAAGTGAA--CAGCT---AAAGGGTGTGCCAATTCTTTTAAGGGATTTGAAGGA-
human    : GTGACCAGCCTCATGGACTGGACTCACAAGCATTCTGGATGGAGACATCTCGGAAGGAATATACCAACTCTTTTGTAATATTTGATGAG-
dog      : GCACCCTGCTCCC---CTCTGCCTCCCCTCTACCTCCCATTCCTTCCTAT----TAGAGCATA--AACGCTATTAGACCATTAAAAGAAA
opossum  : NNNNNNNNNNNNNNNNNNNNNNNNNNNNNNNNNNNNNNNNNNNNNNNNNN-----NNNNNNNNNNNNNNNNNNNNNNNNNNNNNNNNNNN
platypus : ACATGGACTCATAGTCTAAATCCCTATCCAGTGCTTAGTATGGTGCTCC-----AGTGCTTAATATGGTGCCTGGCACCTAGTAAGCAT

mouse    : TTTCCATGTAGAACAAAGCCAGAC--GAGTTGTCTGTGATTCTGGGGCTCTCAGCAAGCAGTAAGTGATGATTACAGAGTGTTTGCCTT
human    : TTTCCACACAGAAGCAGTGTTAGACCTAAATTTCCTATGGTTCTGGAGAGCACAGCAAAAACTAAATGATGGCTCCAGAGCAACAGCATT
dog      : TTTCTCCCAAGAAAGTTCGTCAGAATGGGCCCCAACACAGAACAGCAACTAACAACAGAAAACGCCCAAATTGGGGGAAATTACTACATT
opossum  : NNNNNNNNNNNNNNNNNNNNNNNNN---NNNNNNNNNNNNNNNNNNNNNNNNNNNNNNNNNNNNNNNNNNNNNNNNNNNNNNNNNNNNNN
platypus : GTAACAAATACCATAATAATTATTAT---TTTACAGATGAGATAAGTGAGGCCTAGAGAAGTTAAGTGACTTTCCCAAAGTCAC-ACAGC

mouse    : TGACAAAGTGAAAAGGAGAAATGTCTGCTAACCATAGCTGTCTGAACAC-------------------------------TCCCTCCCGA
human    : TAATTCAAAT-AAAAGAAGAAATCTCTATCAACCAGAATGATCTGAAAACGAAATGGACTACCTAGTTGAGTAGTGAGTCTCCTGTCCAA
dog      : TGAGTACAAT-----TTGCAAATCATCTTGGGAGAGAGTCTTCCCGCAAAGTGAGAGCCAATTTTAAAACACCCACATCCTCTGGCCTCA
opossum  : NNNNNNNNNNNNNNNNNNNNNNNNNNNNNNNNNNNNNNNNNNNNNNNNNNNNNNNNNNNNNNNNNNNNNNNNNNNNNNNNNNNNNNNNNN
platypus : AGACTAGTGG-CGGAGTTGAGATTAGAACCCAGGTGCTCTGACTTCCAGACTCATGCTCTATTCATTAGGCCACACTGCTTCTTTTAAAA

mouse    : CCCCCACTCCGA----------ACCTTTCCTCCCCCCCCCCCC-----------CGCCCCCCGCCAG-------------------CTGA
human    : GCCTTACCTGGGTATGAGTTGTATATTTCATTCTATTACACATATAATAAGGGACGTTTCGTAGGTAGGCTATTCCTGAGGAAGGTCTGG
dog      : CTCCCTCCTCCCCA--------AAGGTTCATCTGGCCTAGCTC--GGCAAAAATCCCTTTGCCTTCATTTGCATTGTTCTTGGGAGGAGG
opossum  : NNNNNNNNNNNNNN--------NNNNNNNNNNNNNNNNNNNNN-----NNNNNNNNNNNNNNNNNNNNNNNNNNNNNNNNNNNNNNNNNN
platypus : TTTTAGCTCTGTTTTCAAG---AAAGGTCTCAGGTTGAGGTGC-----AAACTTCCACATTCAGTTATTTCTTTCAGACTCAAGCAGGGC

mouse    : ATACCCAG---CAGGGGA--TTGAGTTGCCTCACCCCA----------------------------TCTTTGGTGGAGTATGAGCTT--
human    : ACACTGAGAAGTAGGGAA--AAGGGATACTCTAATACAAATAAAATTAAACATCA--TTAGTAGCTCCCTATGTGGGTAATGAGCACTT
dog      : AAAGTGAG---CGAGCCC--TGCAGTCACTCCCCCTGAGAAGACAGTCAGAATCAC--ACCTT---CAACTCAATCGGCCGTGACTTCTT
opossum  : NNNNNNNNNNNNNNNNNNNNNNNNNNNNNNNNNNNNNNNNNNNNNNNNNNNNNNNN----------NNNNNNNNNNNNNNNNNNNNNNNN
platypus : ATATCTGGGCTGAAGTGACCTAGAGGGGCCCCATTTCTTTAAATATCTCCCTCCCTTGATTTTATATGTCATGTTTAGGAGTGTGTTGTG

mouse    : ------------------GGCCTGAGCTGTGTGTTCTATT-------CTAGGATATACATAATG---GGT--------------GCTGTG
human    : CCATGTACCAGCACAACAATCCTGTGCAGTGAGTCCTATAATTACCACCATGATACAGGCAAGG---AGACTGAGATGGAGACAGGTGTG
dog      : GGGAAT-TTGGCTGAAGGGTCCCAAAC---GAGTCAAACTTAATTAGTCAACTTTTACCCAGA---GCATGTAGACACTTGATGCTTTG
opossum  : NNNNNNNNNNNNNNNNNNNNNNNNNNNNNNNNNNNNNNNNN-----NNNNNNNNNNNNNNNNN---NNNNNNNNNNNNNNNNNNNNNNNN
platypus : GGGTGGGAGAGATTGATAGCTTTCGCTGGCCCATTGCATTTA-----TTTATTTGTACATAATGCCAGATTTTTTTAAAAAAGAGCAGTA

mouse    : TGAGT------------------------------------------------------------------CTGCTATTCCAAAGGA---
human    : TAACTTGTCCAAGGTTGCACAGCGAGGAGGTGCAGAGCT-GGGACTCAAATCCTAGCAGCCAAACTACCACCCATTATGCCAGAGTACCC
dog      : TAAGAAAATGAGTGCACCATACCCGTTGACAAGAAAATG-TGGGTGCTGGTCACCAAAA-------------AGAAAATAGAAAGGGGAA
opossum  : NNNNNNNNNNNNNNNNNNNNNNNNNNNNNNNNNNNNNNN-------------------------------NNNNNNNNNNNNNNNNNNNN
platypus : GCAGACATGGAGTGGATTTTTCTATATACTATGAGAATGCTTAGCTAGCATAGCCTCAATTCTGGGGTCCTATTTTGCCTGCAAGAAATG

mouse    : -----------------------------------------------------------AGGTCTTGACACAG--------GAGCAGGGA
human    : GCTCCATTCCTACTTCCTCTCTACCTCCCCAGTCCCTTCTATTATAGTATAAAACGACTAGAGCTTGAAAAAGAGATTTCTGGACAGGCA
dog      : GAGTGGTGGTTATTTATTTATAAAGACTTTATTTATTTATTTATTCATGAGAGATACGGAGAGGCAGAGACACAGGCCGAAGGAGAAGCA
opossum  : NNNNNNNNNNNNNNNNNNNNNNNNNNNNNNNNNNNNNNNNNNNNNNNNNNNNNNNNNNNNNNNNNNNNNNNNNNNNNNNNNNNNNNNNNN
platypus : AGGGGAATTGAAGTCAGAGAGGAAAAGGTAAGTGGAAATAGAGGTCTAGGAGAGCTGGAACTTCTACGTTTAATTAGGTGTGAGAATAAA

mouse    : AGGATGCTTA------------GCACAACTTAAAA-----ACCATTTAATAGCTCAATATG----------TTGGCT-CTGAGCATCTAT
human    : CGGTGGCTCACGCC-------TGTAAATCCCAGCACTTTGGCATTTTGGGAGGCCAAGACAAGAGGATTGCTTGAGC-CCAAGAGTTCGA
dog      : GGCTCCCTGTGGGGAACCTGATGTGGGACTCGATCCCAGGATCACGCCTGAGCCAAAGACAAA-----CACTCAACCACTGAGCCACCCA
opossum  : NNNNNNNNNNNNNN-------NNNNNNNNNNNNNNNNNNNNNNNNNNNNNNNNNNNNNNNNNNNNNNNNNNNNNNNNNNNNNNNNNNNNN
platypus : ACTAAGGTCCTAATA---TTTAGGGGAACATTAAAATAGACTCCACGCTGGGTGCCATTTAAGAGGAGAGTGTGAATGATATTACAGTCA

mouse    : GTTCA-CTGAACGCCATAGC-----CCTGT----ACAGA-AGCCTGATAGTTATCACCATGCTCTAGA----TGAGGAAACAGAT-TGTA
human    : GACCAGCTGGGCGACATAGTGAAAACTTGTGTCTACAAA-ATAATAATAATAATAAAATGCCGGGCA----CGGTGGCTCACACCTGTA
dog      : GGTGTCCCATGTTTCTTCCTTTTAATTGGATGTAATTGATATTATACTAGTTAACATCATATTGGTTAACAGTATAGTAGTCGACATATT
opossum  : NNNNNNNNNNNNNNNNNNNNNNNNNNNNNNNNNNNNNNNNNNNNNNNNNNNNNNNNNNNNNNNNNNNNNNNNNNNNNNNNNNNNNNNNNN
platypus : GTCAGTCAATCGTATTTATTGAGAGCTTACTGT-GTGCAGAGCACTGTACTAATCACTTGGGAGAGTAA-AATAGAAAAATAGACACATT

mouse    : C-------------------------AGGTAACTAACTCATCCAAGGT---CA------------CACAGCTGGAAGCACGG-----AGC
human    : ATCCCAGCTCTCTGG----GAGGCCGAGGCAGGTGGATCACTTGAGGT---CAGGAGTTCGAGACCAGGCTGGCCAACATGG--TGAAAC
dog      : CTATAATAATACAAG----ATAGTAACGACATTATAGTAGTTGCAGGTATACAACAGCATGATTTGATATTTGTACGTATTG--TGAAAC
opossum  : NNNNNNNNNNNNNNN----NNNNNNNNNNNNNNNNNNNNNNNNNNNNNNNNNNNNNNNNNNNNNNNNNNNNNNNNNNNNNNNNNNNNNNN
platypus : CCTTGCCCACAACGAGCTTACAGCCTAGAGAAGCAGCGTGGCTCAGTTGAAAGAGCACGGGCTTGGGAGTCAGAGGTCATGGGTTTGAAC
```

```
mouse    : CAGGACTCTGCTCCTCATGGCTAAATTGCCT-----------TCCACCCTTCTCCCCTCAGGCTTTCAAA-------------------
human    : CTTGTCTTTACTGAAAATACAAAATTAGCTGGGCGTAGTGGCGCACACCTGTCATCCCAGCTACTCAGGAGGCAGAGGCACAAGAATCG
dog      : ---GACCACGGTAAGCCTAGTTAACATCTGTCATCATGTATAATTACAGATTTTTTTCTTGCTTTTATTTTATTTATTG------ATCTA
opossum  : NNNNNNNNNNNNNNNNNNNNNNNNNNNNNNNNNNNNNNNNNNNNNNNNNNNNNNNNNNNNNNNNNNNNNNNNNNNN-----------
platypus : CCCGGCTCTGCCACTTGTCAGCTGTGTGACTGGGGGCAAGTCACAACTTCTCTGTGCCTCAGTTACCTCATCTGTAAAATGGG-------

mouse    : ----------------------TGCTACCTCCCTGCTTCCTTCTA-----TTCAAGCGTAGAAACCA-GAGC-----TTT---------A
human    : CTTGAACCCAGGAGGCGGAGGTTGCAGTGAGCCAAGATCCTGCCACTGCATTCCAGCCTGGGTAACA-GAGCGAGAACTTGTCTCAAAAA
dog      : TTTATTAATTTTTAGTTAAGTAAGCTGTACGCCCAA--CGTGGCGCTTGAACGGACCCCGAGATCAA-GAGTCGCATGGTCTGCTGACTG
opossum  : ---NNNNNNNNNNNNNNNNNNNNNNNNNNNNNNNNNNNNNNNNNNNNNNNNNNNNNNNNNNNNNNNNNNNNNNNNNNNNNNNNNNNNNNNN
platypus : ---ACTAACTGTGAGTCTCATGTGGGACAACTTGATTACCCTGTATCTACCCCAGGGCTTAGAACAGTGCTCTGCACATAGTAAATGCTT

mouse    : AAAAGTGACTTCTACCAAGAACGTTCAGCAGAATGAGCCC----AACATAGAACGACAACTCA-CAGCGACACAAAAGGCCCAAACCAGA
human    : AAAAAAAAATTCTACCAAGAAAATTCATCAGAATGGGCCCCA--AACTCAGAATAGTAACTAA-CAAT--TAAAAAAGACCTAAATCAGG
dog      : AACCAACCAGGCGTCTCACAGATTTTTTTTGTGTGATGAT----AACTTTTAAGATCTACTTATCAAT--TTTCAAATGCACAACACAGT
opossum  : NNNNNNNNNNNNNNNNNNNNNNNNNNNNNNNNNNNNNNNN----NNNNNNNNNNNNNNNNNNNNNNNNNNNNNNNNNNNNNNNNNNNNNNNNN
platypus : AACAAATACCAACATTATTAATATT-ATTAGAGGGGGTACGGGCAACGAGCAAGGGTCAGTTCTTGGCCCTCTTCTGTTCTCCATTTACA

mouse    : GGAAATTACCCTGC------ACAATTTGCAAATCACCTCAACAGAG--------TCTTCTGGGCAAAATGAAGGCTAATTTTAAAACATC
human    : GGAAATGACCATGCTTGAGTACAATTTGCAAATCACCTTGAAAGAGAGAGTATGTCTTCCAGA-AAATCGAGAGCCAATTTTAAAACACC
dog      : AGCATTAACTCTAGTCA---CCATGCTGTACATTACATCCCCAGGACTTATTTATTTTATAACAAGAGTTTGTGCC--TTTGACCACTTT
opossum  : NNNNNNNNNNNNNNNNNNNNNNNNNNNNNNNNNNNNNNNNNNNNNN--------NNNNNNNNNNNNNNNNNNNNNNNNNNNN----NNNNN
platypus : CTCACTCCCTCGGTGAACTCATTCGCT-CTCATGGCTTTGACTACC--------ATCTCTATGCAGATGACACGCAGATCT----ACATC

mouse    : CACATCCTCTGGGCTCCGCCCTTCCCCCCCACCCAGAGGTTCT--TGGGGCCAT---TCAAGCAAAAACCCCTCTGCTCTCATTTGCAT
human    : CACATCCTCTGGGC------CTCTCCCTCCTCCCCAAAGTTTCT--TCTGGCCTTG-CTCAGGCAAAAATCCCTTTGCCTTCATTTGCAT
dog      : CGCCCGTTTTGCCCACC-CTCTATCCCTGCCTCTGGCGACCACCGATCTGTTCTTG-TATCTATGAACTTGGGTTTGTTTGTTCTTAGAT
opossum  : NNNNNNNNNNNNNNNNNNNNNNNNNNNNNNNNNNNNNNNNNNNNNNNNNNNNNNNNNNNNNNNNNNNNNNNNNNNNNNNNNNNNNNNNNNN
platypus : TCCGTCC-CTGTCCTCTCCCCTCCCTTCAGGCTCGCATCTCCTCCTGCCTCCAGGATGTCTCCACCTGGATGTTGGCCCGCCACCTAAA

mouse    : TGTTCTTGGGAGGG-----GGAAAGTG---AGCAAGCTCTG---AGGTGTCACTCCCCTGAGAGGTGCAGCAGGGTCACACCTCGTTTGG
human    : TGTTCTTGGGAGGG-----GGAGAGTG---AGCCAGCTCTG---AAGT--CACTCCTCTGAGAAGAGAGCCAGAATCACACCACAGTCAG
dog      : TCCACATGTAAGTGAGATCGGACAGTATTTGTCTTTCTCTTTCTGACTTACTGTACTTAGAATGATGCCTTCAAGATCCATCCGTGTTGT
opossum  : NNNNNNNNNNNNNN-----NNNNNNNNNNNNNNNNNNNNNNNNNNNNNNNNNNNNNNNNNNNNNNNNNNNNNNNNNN----NNNNNNNNNNNN
platypus : ACTCAACATGAGCA-----AGACTGAGCTCCTCATCTTCCCTCCCAAACCCGGTCCTCTCCCAGACTTCTCTA----TCACCGTGGATGG

mouse    : CACAACCGGATCAGGCGTGAAC-CTGTGCAAAGTTGGCCTA-----CAGTCCAAATGAGT-TAA-----TTAGTCAGCATTGCC-----C
human    : TGTGACCAG------CGTGACCTCCGTGGGAATTTGGCTGAAG---CATCCAAAATGAGT-CAAATGAATTAATCAACTCTTCC-----C
dog      : CACAAATGG-CAAGATTTCCTCCTTTTTTATAGCCAAATAATATCCCATTATATATATATATAATAATATTAATATATATAAATAACAAC
opossum  : NNNNNNNNNNNNNNNNNNNNNNNNNNNNNNNNNNNNNNNNNN----NNNNNNNNNNNNNNNNNNNNNNNNNNNNNNNNNNNNNNNNNNNNNN
platypus : CACGACCATCCTTCCCGTCTCTCAGGCCCGCAATCTCGGTGT----CATCCTTGACTCGTCTCTCTCGTTCACCCCACACATCCGATCTG

mouse    : CCAGAGAAGGTAG-----ACGATTGACCTTTCCCAAGAAAATGAACGTGCCCCACCCAGTGA-CAAGAAATGAGGGCACCGGTCAT-GCG
human    : CTAGAGAATGCGG-----ACACTTGATCCTTCACAAGAAAATGAGGATGCCCCACCCAGTGG-CAAGAAATGAGGGCACTGGTTACCAAA
dog      : TCAGGGCGCCTGGGTGGCACAGTTGGTTAAGCACCTTACCTTGATTTTGACTCAGGTTATGATCTCAGGGTCATGAGATTGAGCCCCACT
opossum  : NNNNNNNNNNNNN-----NNNNNNNNNNNNNNNNNNNNNNNNNNNNNNNNNNNNNNNNNNNNNNNNNNNNNNNNNNNNNNNNNNNNNNNNNN
platypus : TTACCAAGACCTG-----TCAGTT---TCACCTCTACAATATCGCCAAGATCCGCCCTTTCCTCTCCACCCAAACGGCTACCTTACTGCT

mouse    : ATAAAAACACA----GGCCTGAGAGAGCTATAGT----AAGAGCTCCTGCTGTGTTGGACCCTGTGCT---GTGG-----------GGT
human    : AGGAAAATAGA----AAAATGGGAAGAACAGTGTTTTAAAGTGCATCTACCTTGTTTGACCCTGAGCTTGGGTGGCAGACCCAGGTGGGT
dog      : TGGGGCTCTGG----ACTCAGCAGGGAGTCTACTT--GAGATCTTCTCTCTTCCTCTGACCCTCCGGT-GCTTGCTTGCTCTCTCTCTAA
opossum  : NNNNNNNNNNN----NNNNNNNNNNNNNNNNNNN----NNNNNNNNNNNNNNNNNNNNNNNNNNNNNNNNNNNNNNNNNNNNNNNNNNNNNN
platypus : ACGGGCTCTCGTTATATCCCGGAGAGACTACTGT----GTCAGCCTTCTCTTTGACCTCCCTTCCTCCTCTCTCGCCCCGCTC-CAGTCT

mouse    : GTGCTGGGC-ACCATCATG-ATCAAGGT----GCATGACCCCTGGAGATTTGGGGAGGC--CCTGTATGGTGAAGTGTGGCAGCTGAC-A
human    : CTGCCAGGCCACCATCATTTGTCAAACT----ACATTCACCCTGGACATTCAGGAAGGC--CCTAAAACTGAAACAGCATCTCCCAAC-A
dog      : AAAAAAAACAATAATAATTTAAAAAAATAAGAAAACATCTCTATCCATCCATCCATCCATCCCTCGATGGACACTTGGGCTACTTCC-A
opossum  : NNNNNNNNNNNNNNNNNNNNNNNNNNNNNNNNNNNNNNNNNNNNNNNNNNNNNNNNNNN--NNNNNNNNNNNNNNNNNNNNNNNNNNNNNNN
platypus : ATTCTTCACTCTGCTGCCCGGCTCATCTTCCTGCAGAAATGATCTGGGCATGTCACTCC--CCTTCTTAAACAACTCCAGTGGTTGCCTA

mouse    : GCATTTGGGAAAC-ATGGAGAATTTCCCCAGGTTTATAC--------------------------------AGCAGTTCACT-CATCTAT
human    : GCATCTGGAGGACCACAAAGAATTTCTGTGGGGCTACATCATTTTCCCCAGTGTTTCTCTATGCCTTGCAAAGCAGTTGATTATGAGGAT
dog      : -TATCTTGGCTATTGTGAACAATGCTGCAACGAACATAGGGGTAT---------------ATTTATTTTCAAGTTATTGTTTCTATTTCT
opossum  : NNNNNNNNNNNNNNNNNNNNNNNNNNNNNNNNNNNNNNNNNNNN---------------NNNNNNNNNNNNNNNNNNNNNNNNNNNNNNN
platypus : TCAACCTCCGCTCCAAACAAAAACTCCTCACTCTAGGCTTCAAGGCTCTCCATCACCTTGCCCCTTCCTACCTCTCCTCCCTTCTCTCTT

mouse    : TTTAAAATCCTGTTTATTTATTTAATGTGTGTGTG---TGTGTGTGAACATCTGT-------GCTGCTGTGTATGTATGTGTGTGGAGAT
human    : CCTAGGCTCTTTTTTTTTTTTTTTTTTTTTTTAAGGGATGGGGGTCTCACTCTGTCTCCAAGGCTGGAGTGCAGTAATGCAATCATAGCT
dog      : TTTGGGTAAATACGAGTGGAATTGCTATCTTTAGC--ATTTTGAAAAACCCCCATACTGTTTTCCATAGTGGCTGCACCAATTTACATCC
opossum  : NNNNNNNNNNNNNNNNNNNNNNNNNNNNNNNNNNNNNNNNNNNNNNNNNNNNNNNN-------NNNNNNNNNNNNNNNNNNNNNNNNNNNNNN
platypus : TCTACCGCCCACCCCGCACGCTCCGCTCCTCTGCCGCCCACCTCCTCACCGTCAC-------TCGGTCTCGCCTATCCCGCCGTCGACCC

mouse    : CAGAGAA----CAACTTTTGGGAGTTAG------TTCTCTCCT---TCCACCA-----------------TATGTGCCCCGAGGATTAGA
human    : CACTGCAGCCTCAAAATCCTGGGCTCAGGGGACCTTCCTGCCTCAGTCTTCCAAGTAGCTGGACTACAGGTATGTACCACCATGCCTAGC
dog      : TGATGTTTTAACACCCCATTGGACTTGGAGCTCCGGGCACCATAGTCAGTCCAGGTCCCTGGGCCA----CCTTCCCCCGTCAGACCACT
opossum  : NNNNNNNNNNNNNNNNNNNNNNNNNNNNNNNTTTCTTTCTTTCTTCCTTCTTTCTTTCTCTCTTTCTCATTCTCTTTCCTTCTTTCTCTCTT
platypus : CTGGACCACGTCCTCCCGCGGTCCTGGAACGCCCTCCCTCCTCACCTCCACCAAACTGAT----------TCTCTTCCCCTCT-TCAAAA

mouse    : CTCTTCATCAGGCTTGGCAGCAAGTG---------CATT----TACCCA--CTG-AGC----------------------------CATC
human    : TATTTAAAAAAATTTTTGGAGAGATGGGGTCT---CACTATATTGCCCAGGCTG-ATCTTGAACTCCTGGCCTTAAGGTATCCTCCCACC
dog      : TTTACCCTGAGATTCAGGGACGTCCTCACCCTGGGAAATGTTGCCTCCCGACTGTAGCCGAGAGCCACCGGGAATCCGTGTGGGGCGAGG
opossum  : TCCTCCCTTATTTCTTTTTATTTCTTC--------CTCTTTTTTTCTTTTTCTTTCTTTCTTCCTTTCTTTCCTTCTGTCTTGGAATCAA
```

```
platypus : CCCTACTTAAAACTCACCTCCTCC-----------AAGAGGCCTTCCCAGACTG-AGCTCCTCTTCTCCCTCTACTC-------CCTCTG

mouse    : TCACTGACCGAAGA----------GCAG----------------TTTATTA------TAGTGATCATGAATGTCCCAC-A-------TAT
human    : TCAGCCTCCCAAAATGCTGGGATTGCAGGTGTGAGCCACCGCCCTCTGCCAAGGATCTGAGGTTCGTGAATATCCCAG-AGCCCTGATAC
dog      : TCATTCTCCAAAGGCCTTCTCTGTGCCCTGCAACGC----GGTTTATTATAAGGATCTGAGGTTCCTAAA-ATCCTAG-CGCCCTGGGAT
opossum  : AGACAGAAGAGCAGTAAGGGCTAGGCAATGGGAAGTAAG-TGATTTGCCCAGGGTCACACAGCTAAGAATTGTCTGAGATTAGGACCTCC
platypus : CCACCCCCCTTTTACCT---CTCCGCAGCTAAACCCT----CTTTTTCCCCCTTTCCCTCTGCTCCTCCACCTCTCCC-T-------TCC

mouse    : GACCCTGAGGTCAC--TGT-ACCAGCATTTCCCAACGCCTTC---CCTTGAGTCACTTTGGGACTTG-TCTGT-------GGGTCTCTGT
human    : AATCTAGAGGTCACATTGT-ATTAACTTTTTCTAACTCCTTCTTCCTTTGAGCCACCTCATGAAGTG-CCTACTTACTTGGGGTTTCCAT
dog      : GATCTAGAGGTCACGCTGT-ATTAACTTTTTCTAATTCCTTCTTCTCTTGAGCCGCCTCACAGAGTGCTTTGTCGTCTGTGGGTCCTTGT
opossum  : CATCTCCACACCTAGTTCTCAATCATCCATTGAGTCATCTAGTT-GTCCCCCCTCTAGTTTTCTTTAAATGATGAGT-GTTTATTCTTGT
platypus : CATCCCCACAGCAC--TGT-ACTCGTCTGCTCAACTGTATAT---ATTTCCATTACCCT---ATTTATTTTGTTAATGGATTGTACATTG

mouse    : CTTAGGT-----------------CAATCCCCAAACATTCCTTGTCCC---AGAGTCCTGGGGATGGTC-AGATAATCATGACTTTTTTT
human    : TCTAGGCTGC-AGGGG-TCCTACCCATTCCCACATCATCACCTTTCCACCAAGGAATCTGAGGGTGACCTGGATAGTCATGATTTTTATT
dog      : TCCAGGCTGC-AGGGGGTCCTGCTCAATCCCAAATCATCCGTTCCCCACC-AGGGGCATGGGG-TGGCCTGAATAGCCATGACTTTTGTT
opossum  : CATGGGTTCCCATGTCAGAGAGAGTTTTTCTTTGACTGGGCCAAATCCCC----ACTTAATGAGAGGGAAAAGAAATTTAGTTGATCATT
platypus : CCTTGATTCT-ATTTAGTTGCCATTGTTTTTACGAGATGTTCTTCCCCTT---GACTCTATTTATTGCC--ATTGCTCTTGTCTGTCCGT

mouse    : TTTTTAATCAGGAGAATTAAAATAATTTTTACCTCCAGGAAATTTCCAAACCAGG---CAAAATGTTCTTG------GCTTTCCACGGC-
human    : T-----AGCAAGACAGTGAAAATAATTTTCATCTCAATGAAATTTTAAGACCAAG---CAGATTGTGCATA------GCCCTCCTTGGC-
dog      : T-----AGCAGGAGAATGAAAGTCATTTTTATCTCCGCGAAATGTC-AAGCCGGG---CAGAATGTGTGTG------GCTCTGGCTGGC-
opossum  : C-----TGTTTTAAGTCAAAAATTTTACCCTGTTGCAAAATTCTTGTTAAGGCACAGTTGAGATGCTTTCTT-----TCTTAGGATCATA
platypus : CTCC--CCCGATTAGACTGTAAGCCCGTCAAACAGCAGGGACTGTCTCTATCTGTTGCCAACTTGTTCATTCCAAGCACTTAGTACAGT-

mouse    : CTCCTGGGGGCCCCGGGCTCTGTGTCCCTG---AGCTGTAGGGAGGATAGCATGCCTGTGTGCAGTG-GGGCTATTCGTCATCAGAACAA
human    : CTCCTGGGACCCCAGGCCACTGTGGCCCC----ACTAGCAGGGAGGAGAGGATGACTTTGCAATGTAAGGGCTATTCGTCATTAGAACAA
dog      : CTCCTGGGCCCCCCCACCCCCCCAGGCCTCCGTGCCCCCCCACCCCCCAGCGTGACTCTGCAGCGTGAGGGCCATCCGTCACTGGAACAA
opossum  : GATTTAAAGCTGGAAGGAACCTTGGAGTCA--------TCTA-GTCTGAGCTCCTCTTTTTATAGAG-GAGAAAATTGAGGCCCACAAAA
platypus : GCTCTGCACATAGTAAGCGCTCAATAAATACTATTGAATGAATGAATGAACTCAACATTTCATATTGGGAAGGAAATGTTTTCTAAATAG

mouse    : --------CAAGGCCAGC--AGCAGTGTGGCTCTCACCTTAT----CAGGGTGGTTGGCCTAGCTGGATTCTATT---AATTGCTGAAGG
human    : --------CAAGGCCAGC--AGCAGCATGGCTCTGGCCTTAT----CGGAGCAGGAGGCCTCGCTGGATTCTATT---AATTGCCGAATG
dog      : ACAAGGCACGAGGCCCTC--GGCAGCGCTGCTCCGGCCTTAT----CGGAGCGGGAGGCCTAGCTGGATTCTATT---AATTGCCCAATG
opossum  : C---TGAGGAGACTTGCCTAAGTAAGGCCTTCTCACATTAGAG--TCACTATTCTTTTCTCCTTATGGAACTGACTC-TCTTCTTAGGGG
platypus : CATGTACATAGCATTAACTTAATGGTGTATCTAAAAAATAATAACATAGTATGAGTTGATAATTTGAGGACCCTTCAGAGTTGTTGGCCG

mouse    : CTGTTGG-----AGTT---GGCAGATA-CAGGA-AACAGTTTACATG--GTGACACAGGGCCCCAGCTCGCAGCCTCTCAG---AAACTG
human    : CTGTTGG-----GTTT---GGCAGATA-CAGGA-AGCAGTTTACATG--GCTACACAGTGCCCTGGCTCACAGTGTCTCTG---AAATGG
dog      : CTGTTGG-----GTTT---GGCAGATA-CAGGA-AACAGTTTACATG--GCTACACGGTGCCCCGGCTCACAGTCTCTCTG---AAATGG
opossum  : ATTTTACCTAGGGGTCCTCCATTTTCCCCAGAGTAAATATAAATTAA--ATTATTTCAAATTCTGCTTGACTAAGGCTCAGGATGGCCTG
platypus : GTCTTAC-----AGCTTGTGATAGATAACAAAACAATAGCCAGCATAAAGCAGCACAGGAAACTGAGGAGTAATCTCTTTGGTCAAATTG

mouse    : TATTATTGTAGTGGCATA---ATTGTGATTAGGGA---CAG--TTCCCTGAGGAAGAGCAGACCTTTGATGATTGAGTCACTTCCTC-TG
human    : TATTATTGTAGTGGGATA---ATTGTGATTAGGGA---CAG--TTCCCCGAGGAAGACCAGGCCTTTGATGATTGAGTCACTTCCTC-TC
dog      : TATTATTGTAGCGGGATA---ATTGTGATTAGGGA---CAG--TTCCCCGAGGAAGACCAGGCCTTTGATGATTGAGTCACTTCCTC-CC
opossum  : TGA-AGCAAGCCAAAGAGGA-TGTGGTCTTTTAAAATTGGCTCTCACTTTATGG--AAAGTCTCCCTCTGTCTGGGTGATTTGCAAACG
platypus : TGGTAGCAATCTGGGAAAGAAAGCATGGCTTTTGA---TGGACTTGATCTGGGAGCAAAAGATTCCCCAC-ATTAAACCATTTGCTT-TT

mouse    : CACTCCACTTCAG-TGTGATAGTGTGGCCC------------CACGGACCCTT--GGCTGGC-CAGAG-ACTTTAGAGTCCCACAGGAAA
human    : TACTCTACTTCAG-TGTGATAGCGTGGCTCGCCCCACAGACACACACCCTT--TGCTGGC-CAGAG-AGCCCTGAGTCCCACAGCAGG
dog      : CACTCGGCTTCAC-TGTGATAGCGCAGCTCGGCCCACAGA--CACACACCCTT--TGCTGGC-CAGGGCGGTCCCCGGGTCCGGGGCCAG
opossum  : CCTTTTACATTAGGTAATTTCCCCTAAGATTTGGGCTTTTGCTGTGTGTTAGGGTTGGACACACACAGGTCCTGGAGGTCCTCAGGGAGA
platypus : CCATATGTTTCA--CAAGATAATATCCATCTCCCATCTTTCATTTGCATCACA--TGCACCC-CATAG--GATATAAATTCTAATTTTTA

mouse    : GCTCTCCAACAGGGCAGTCCAACCTC------
human    : GTTCCCAAACCAAG--AGCCAAGAGCT------
dog      : GCTCCCCGGGTGGG-CAGGAGGGCCTGGGAGCC
opossum  : CCCTTTCTTAAGTCCT-----------------
platypus : TTTTTTCCTCAGTGATGATTTGGACT-------
```

Gata

Ets

Meis

Gfi1

Ebox

Run
